# Supplementary material for: Compounds activating VCP D1 ATPase enhance both autophagic and proteasomal neurotoxic protein clearance
Source: Nat Commun. 2022 Jul 16;13:4146. doi: 10.1038/s41467-022-31905-0 (PMC9288506; doi:10.1038/s41467-022-31905-0)

Supplementary Information

Compounds activating VCP D1 ATPase enhance both autophagic and proteasomal neurotoxic protein clearance

Lidia Wrobel[1,2#], Sandra M. Hill[1,2,3#], Alvin Djajadikerta[1,2], Marian Fernandez-Estevez[1,2], Cansu Karabiyik[1,2], Avraham Ashkenazi[1], Victoria J. Barratt[1,2], Eleanna Stamatakou[1,2], Anders Gunnarsson[4], Timothy Rasmusson[5], Eric W. Miele[5], Nigel Beaton[6], Roland Bruderer[6], Yuehan Feng[6], Lukas Reiter[6], M. Paola Castaldi[5], Rebecca Jarvis[7], Keith Tan[7], Roland W. Bürli[7], David C. Rubinsztein[1,2,*]

Supplementary Fig. 1.

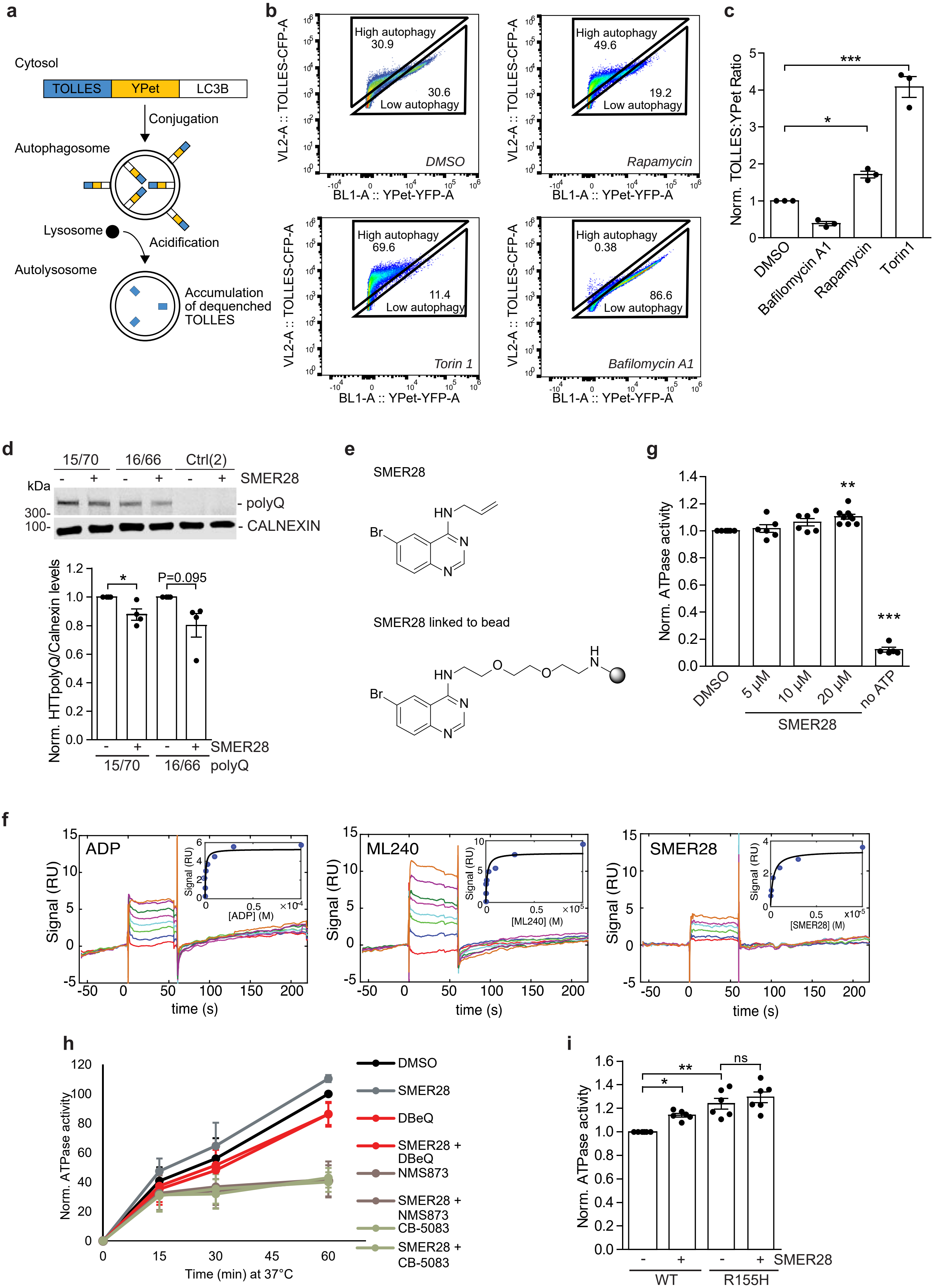

Supplementary Fig. 1. SMER28 binds VCP and increases VCP D1 ATPase activity.

a, Schematic representation of the SRAI-LC3B reporter assay. Delivery of the SRAI-LC3B reporter to lysosomes causes degradation of YPet, which leads to a detectable shift in fluorescence of the tandem construct as the FRET-associated quenching of the TOLLES signal is relieved after YPet degradation. **b, c**, Validation of the SRAI-LC3B autophagy flux assay. HeLa cells expressing SRAI-LC3B were treated with 1 µM Rapamycin, 250 nM Torin 1 or 100 nM BafA1 for 24 h, followed by FACS analysis. The median ratio between TOLLES and YPet was taken for each condition; higher ratios reflect higher levels of autophagy; n=3; one-way ANOVA: $P < 0.0001$ with post hoc Tukey test, Rapamycin $P = 0.0404$, Torin1 $P < 0.0001$. Representative distributions of TOLLES and YPet fluorescence in (**b**) where treatment with autophagy modulators causes marked shifts in the fluorescence distributions of the cells. **d,** Control (Ctrl) and Huntington's Disease (HD) fibroblasts expressing mHTT-polyQ70 or mHTT-polyQ66 were treated with 20 µM SMER28 for 24 h. Cells were lysed and analysed by Western blotting with anti-polyQ antibody, n=4; one sample t test, HD15/70 $P = 0.0497$, HD16/66 $P = 0.0955$. **e,** Structure of SMER28 (top) and the linker-modified analog coupled to beads used for immunoprecipitation and target identification (bottom). **f,** SPR sensograms illustrating the concentration-dependent binding of ADP, ML240 and SMER28 to immobilized fl-VCP. **g,** *in vitro* ATPase activity of VCP upon addition of increasing concentrations of SMER28 in the presence of 2 mM ATP, normalized to untreated DMSO control, n=9; one-way ANOVA: $P < 0.0001$ with post hoc Tukey test. **h,** *in vitro* ATPase activity of VCP upon addition of 20 µM SMER28 with or without 10 µM DBeQ, 10 µM NMS873 or 2 µM CB-5083, n=9. **i,** *in vitro* ATPase activity of wild-type (WT) and VCP-R155H mutant upon addition of 20 µM SMER28, n=6; one-way ANOVA: $P < 0.0001$ with post hoc Tukey test, WT SMER28 $P = 0.0384$, WT vs. VCP-R155H $P = 0.0004$. Bar graphs data presented as normalized mean ± SEM. *$P < 0.05$, **$P < 0.001$, ***$P < 0.0001$; ns, not significant. Source data are provided as a Source Data file.

Supplementary Fig. 2.

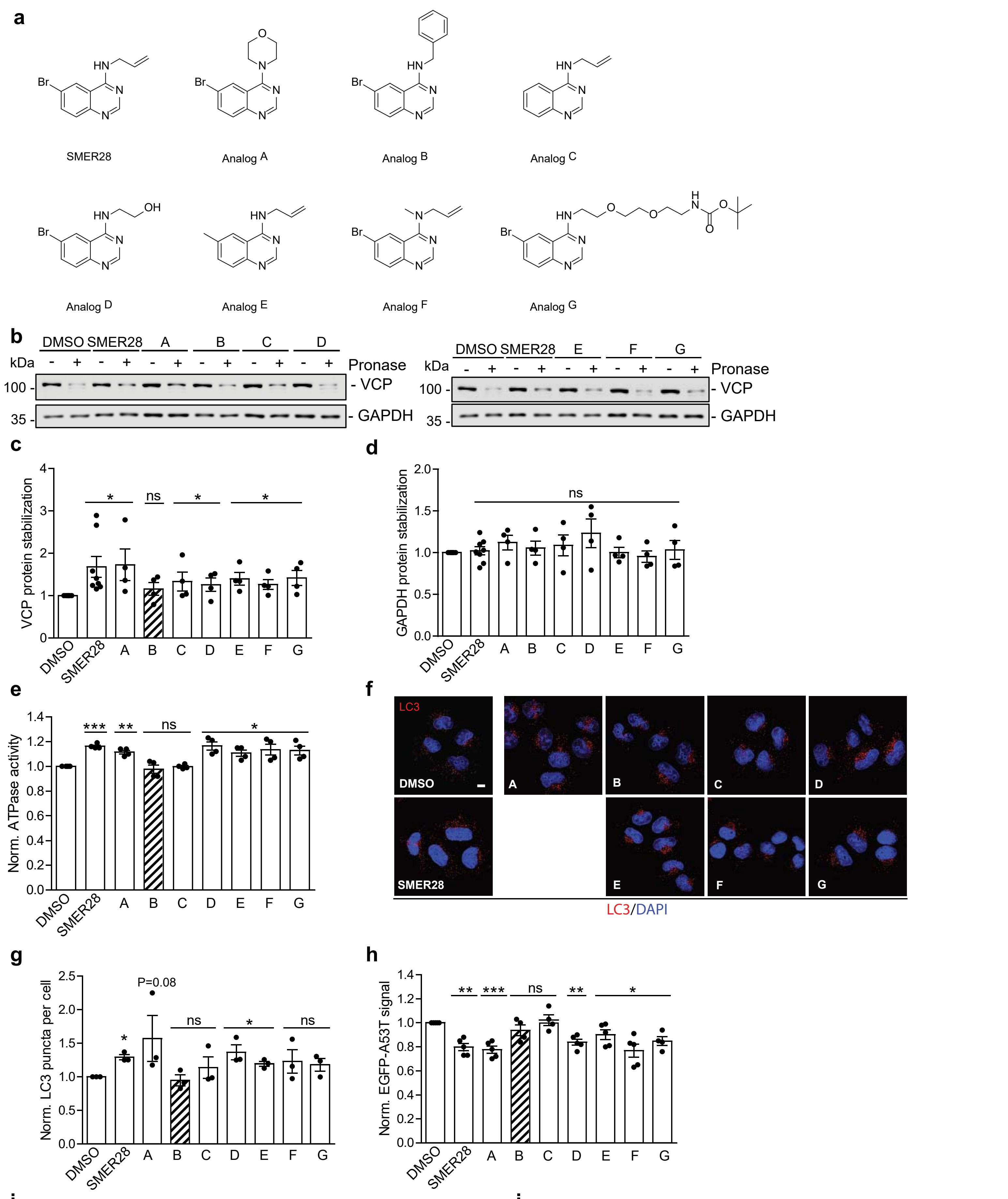

i

LiP Peptide	R^2	EC_{50} (μM)
VTMDDFR	0.955	95.4
EDNSVVSLSQPK	0.930	71.6
REDEEESLNEVGYDDIGGCRK	0.907	63.6

j

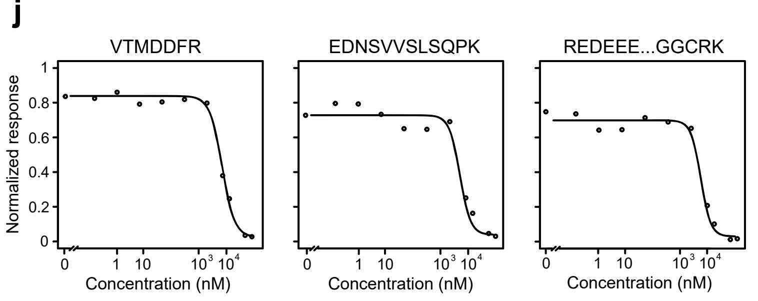

Supplementary Fig. 2. SMER28 structural analogs bind VCP and induce autophagy.

a, Structures of SMER28 and its structural analogs A-G. **b-d,** DARTS assay of HeLa cells treated with 20 μM of SMER28 or analogs A-G. Representative Western blot for DARTS assay (**b**) after treating HeLa cells with SMER28 analogs, 20 μM 1 h, and digesting the lysate with Pronase. Ratio of VCP (**c,** SMER28 P = 0.0149, A P = 0.0155, B P = 0.1405, C P = 0.0471, D P = 0.0311, E P = 0.0024, F P = 0.008, G P = 0.0053) or GAPDH (**d**) levels compared to undigested sample normalized to DMSO sample; n=8. **e,** *in vitro* ATPase activity of recombinant VCP-GST after treatment with 20 μM SMER28 analogs A-G; n=4; SMER28 P = 0.000003, A P = 0.0003, B P = 0.5173, C P = 0.6795, D P = 0.0019, E P = 0.0036, F P = 0.0216, G P = 0.0106. **f-g,** Immunofluorescence analysis of LC3 positive autophagosomes in HeLa cells after 8 h treatment with 30 μM SMER28 or its analogs. Co-treated with 400 nM BafA during the last 6 h. Normalized to DMSO, n=3; representative images in (**f**), SMER28 P = 0.0018, A P = 0.0825, B P = 0.5586, C P = 0.441, D P = 0.0298, E P = 0.0063, F P = 0.2556, G P = 0.1241. **h,** FACS analysis of degradation of mutant α-synuclein (EGFP-A53T) after 24 h treatment with 20 μM SMER28 or its analogs A-G; n=5, SMER28 P = 0.0002, A P = 0.0001, B P = 0.171, C P = 0.5868, D P = 0.0002, E P = 0.0409, F P = 0.0024, G P = 0.0018. Analog B highlighted with diagonal stripes to denote a non-binding and non-functional analog. **i,** The three top-ranked VCP LiP peptides by correlation. Peptides also show the lowest predicted EC50s of all peptides. **j,** Dose response plots for peptides identified in (**i**). Bar graphs data presented as normalized mean ± SEM. *P < 0.05, **P < 0.001, ***P < 0.0001; unpaired two-tailed Student's t-test; scale bar = 10 μm; ns, not significant. Source data are provided as a Source Data file.

Supplementary Fig. 3.

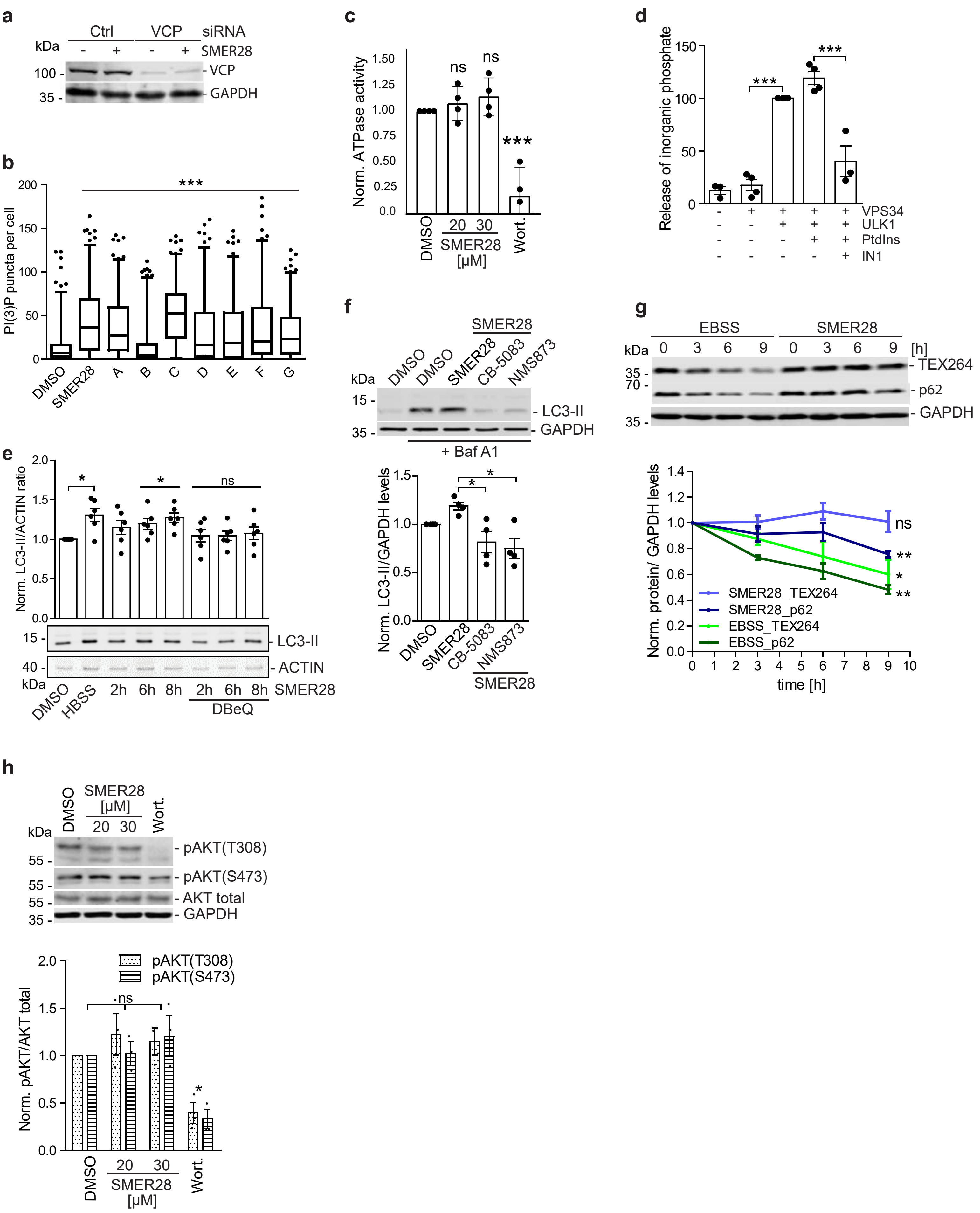

Supplementary Fig. 3. Autophagy induction by SMER28 depends on VCP and PI3K activity, but does not affect VPS34 kinase activity.

a, HeLa cells control or treated with siRNA against VCP were treated with 20 µM SMER28 for 4 h, lysed and analysed for VCP knockdown efficiency, related to Fig. 3e, f. **b,** Immunofluorescence analysis of PI(3)P puncta in HeLa cells treated with 20 µM SMER28 analogs for 4 h; n=3; the box indicates the upper and lower quantiles, the thick line in the box indicates the median and whiskers indicates 2.5th and 97.5th percentiles. *P*-values by a two-tailed Wilcoxon signed rank test. **c,** Intrinsic ATPase activity of VPS34 upon addition of SMER28 or 1 µM Wortmannin, n=4; one-way ANOVA: $P < 0.0001$ with post hoc Tukey test, Wortmannin $P = 0.0001$.**d,** *in vitro* kinase control assay. Purified VPS34-FLAG was pre-incubated for 20 min with ± ULK1 (10 ng/µl) and with 1 µM VSP34 inhibitor when indicated, prior to incubation with Malachite Green for 20 min. Samples containing purified VSP34-FLAG and recombinant ULK1 showed an increase in phosphorylation as expected. The relative levels of released phosphate were determined against a phosphate standard curve, n=4; one-way ANOVA: $P < 0.0001$ with post hoc Tukey test. **e,** LC3-II levels in HeLa cells treated with 20 µM SMER28, with or without addition of 10 µM DBeQ for 2, 6 or 8 h, in the presence of 400 nM BafA1; n=6; HBSS $P = 0.0144$, 6 h SMER28 $P = 0.0366$, 8 h SMER28 $P = 0.0058$. **f,** LC3-II levels in HeLa cells treated with 20 µM SMER28, with or without 5 µM CB-5083 or 10 µM NMS873 for 8 h in presence or absence of 400 nM BafA1, n=4; one-way ANOVA: $P = 0.0076$ with post hoc Tukey test. **g,** TEX264 and p62 levels in HeLa cells treated with 20 µM SMER28 or incubated in EBSS for indicated time points, n=3; paired two-tailed Student's t test, EBBS_TEX264 $P = 0.0254$, EBSS_p62 $P = 0.001$, SMER28_TEX264 $P = 0.9062$, SMER28_p62 $P = 0.0007$. **h,** AKT phosphorylation levels in HeLa cells treated with 20 µM or 30 µM SMER28 or 1 µM Wortmannin for 6 h, n=3, AKT(T308) Wort. $P = 0.0321$, AKT(S473) Wort. $P = 0.0221$. Bar graphs data presented as normalized mean ± SEM. *$P < 0.05$, **$P < 0.001$, ***$P < 0.0001$; one sample t-test unless stated otherwise; ns, not significant. Source data are provided as a Source Data file.

Supplementary Fig. 4.

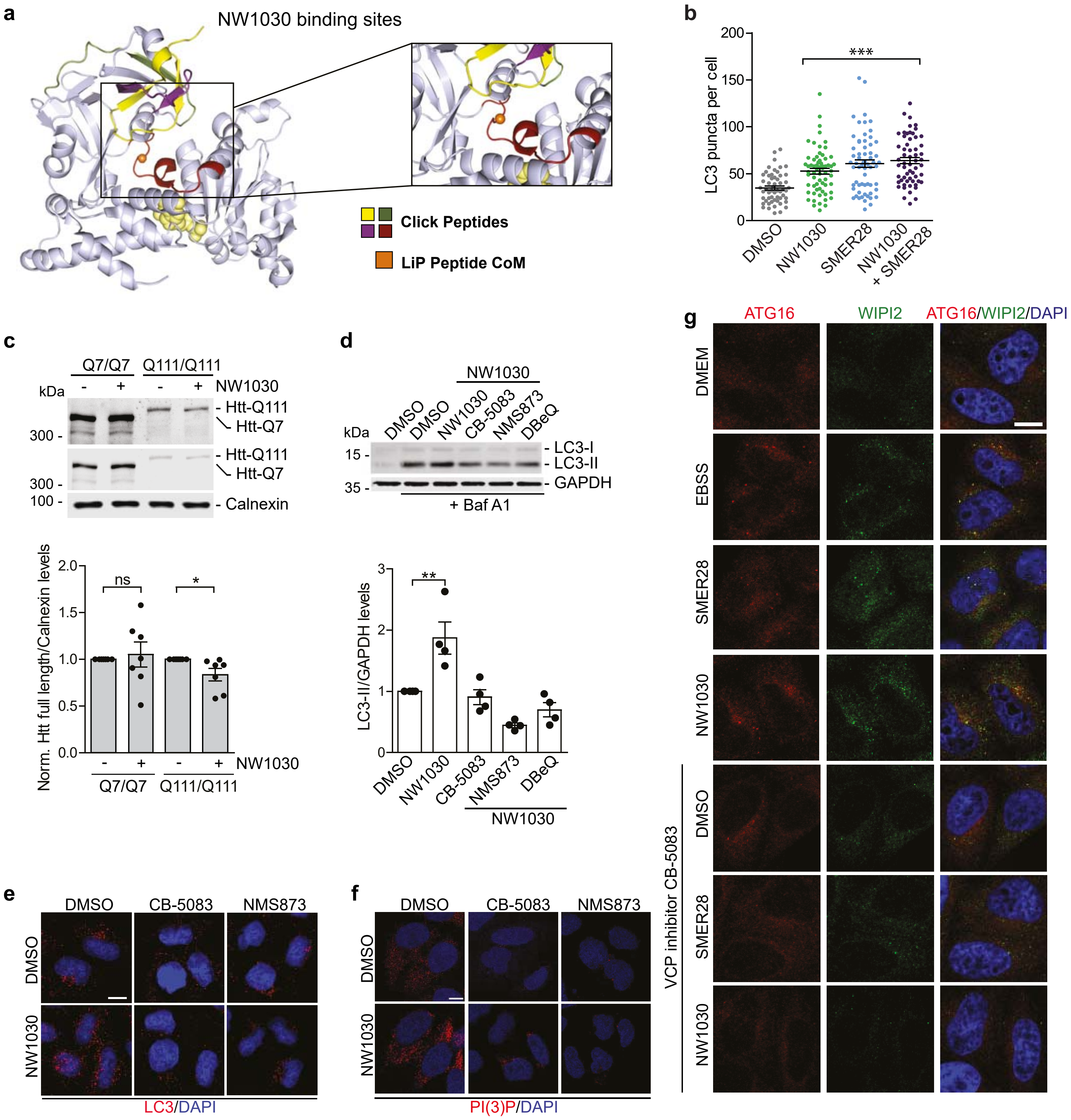

Supplementary Fig. 4. Autophagy induction by SMER28 and NW1030.

a, Peptides from (Figuerola-Conchas *et al.*, 2020; [47]) mapped to VCP subunit with predicted LiP CoM (orange) indicated. **b,** Number of LC3 puncta per cell in HeLa after 8 h treatment with 10 μM NW1030, 20 μM SMER28 or both in presence of 400 nM BafA1; Kruskal-Wallis test: $P < 0.0001$ with post hoc Dunn's Multiple comparison test. **c,** Mouse striatal cells expressing wild-type (Q7/Q7) or mutant (Q111/Q111) huntingtin were treated with 10 μM NW1030. Cells were lysed and analysed by Western blotting with anti-huntingtin antibody, n=7; one sample t test, Q7/Q7 $P = 0.7085$, Q111/Q111 $P = 0.0481$. **d,** LC3-II levels in HeLa cells treated with 10 μM NW1030 in presence of 5 μM CB-5083, 10 μM NMS873 or 10 μM DBeQ where indicated for 8 h; n=4, one-way ANOVA: $P < 0.0001$ with post hoc Tukey test. **e,** Representative images of LC3 puncta in HeLa cells after 8 h treatment with 10 μM NW1030 with or without 5 μM CB-5083 and 10 μM NMS873, quantification in Fig. 4b. **f,** Representative images of PI(3)P puncta in cells treated with 20 μM SMER28 for 6 h with or without 5 μM CB-5083, 10 μM NMS873, quantification in Fig. 4c. **g,** Representative images of ATG16 and WIPI2 puncta in HeLa cells in basal (DMEM), starvation (EBSS 2 h) or treated with 20 μM SMER28 or 10 μM NW1030 for 4 h, quantification in Fig. 4d-f. Bar graphs data presented as normalized mean ± SEM. *$P < 0.05$, **$P < 0.001$, ***$P < 0.0001$; scale bar = 10 μm; ns, not significant. Source data are provided as a Source Data file.

Supplementary Fig. 5.

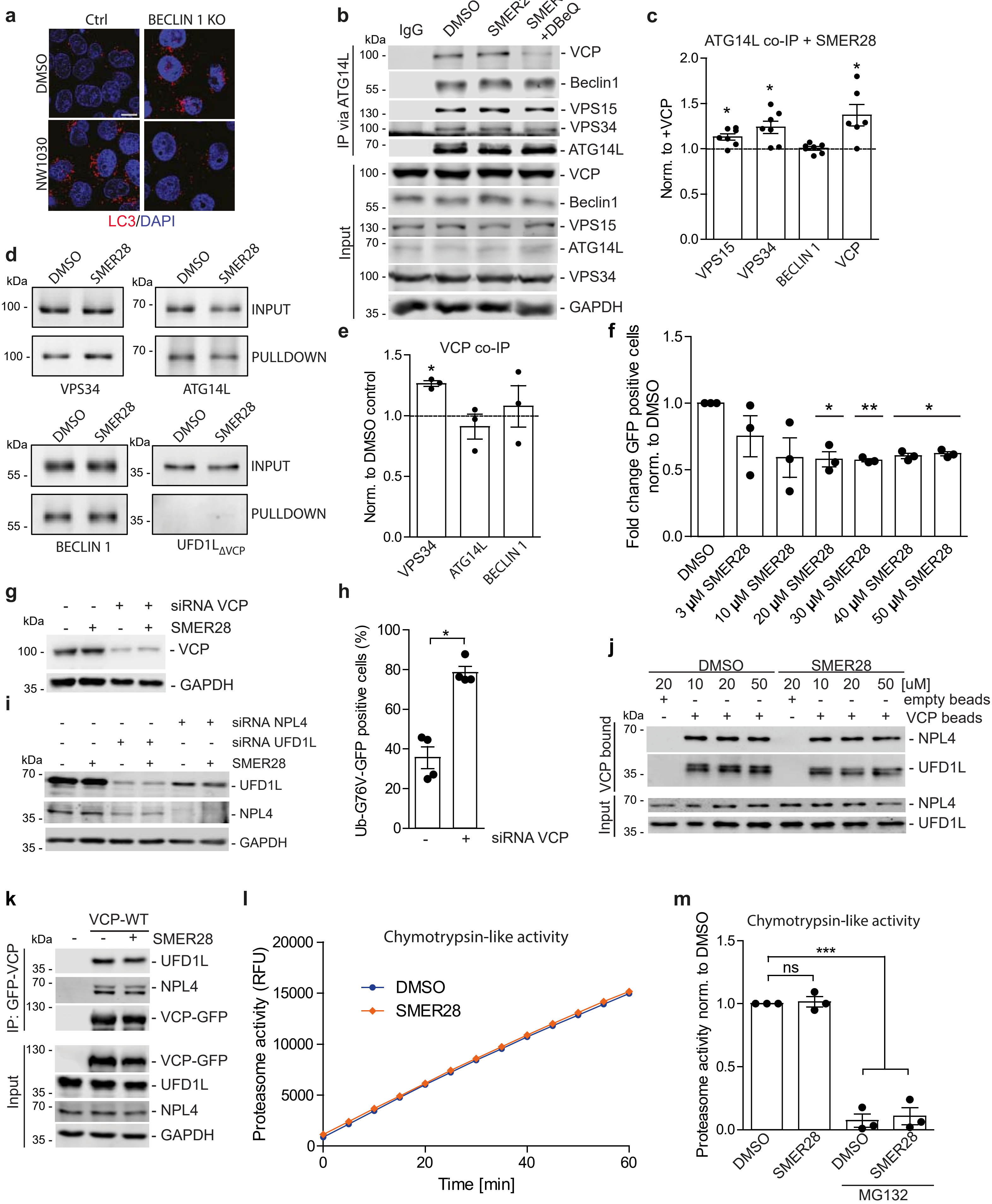

Supplementary Fig. 5. SMER28 enhances VCP stimulation of PI3K complex formation and increases UPS flux.

a, Representative image of LC3 puncta in Beclin 1 knock-out and control cells upon treatment with 10 μM NW1030; quantification in Fig. 5e. **b,** Immunoprecipitation of endogenous ATG14L from HeLa cells treated with 20 μM SMER28 in the presence or absence of 10 μM DBeQ for 6 h. **c,** Increased *in vitro* PI3K interactions by addition of VCP is further enhanced with additional SMER28 treatment. Same data as in Fig. 5h-i, but ratios are normalized to samples containing PI3K components and VCP; one sample t test, VPS15 P = 0.0103, VPS34 P = 0.013, VCP P = 0.025. **d, e,** *In vitro* immunoprecipitation with VCP-GST beads and individually purified PI3K components: VPS34, ATG14L, Beclin 1 and UFD1L$_{\Delta VCP}$ (Δaa215-241) as a negative non-binding control in the presence or absence of 20 μM SMER28, quantified in (**e**); one sample t test, VPS34 P = 0.0078. **f,** HeLa cells stably expressing Ub-G76V-GFP were treated with increasing concentrations of SMER28 for 6 h, followed by FACS analysis; n=3; one sample t test, 20μM P = 0.0176, 30μM P = 0.0007, 40μM P = 0.0025, 50μM P = 0.0018. **g, i,** HeLa cells treated with control or siRNA against VCP (**g**) or UFD1L and NPL4 (**i**) were treated with 20 μM SMER28 for 6 h, lysed and analysed for VCP (**g**) or UFD1L and NPL4 (**i**) knockdown efficiency, related to Fig. 6d, e. **h,** Control experiment showing VCP-dependence of Ub-G76V-GFP degradation. Ub-G76V-GFP cells were treated with VCP siRNA and analysed by FACS after 48 h, n=4; paired two-tailed Student's t test, P = 0.003. **j,** *In vitro* VCP and UFD1/NPL4 binding assay. Purified UFD1/NPL4 heterodimer was incubated with purified VCP cross-linked to the beads in the presence of increasing concentrations of SMER28 or DMSO as control. **k,** HEK293 cells expressing VCP-WT-GFP were treated with 20 μM SMER28 or DMSO for 6 h, followed by immunoprecipitation of VCP-GFP with its binding cofactors. **l,** Representative graph showing no difference in chymotrypsin-like activity of proteasome in HeLa cells treated with 20 μM SMER28 or DMSO for 6 h; quantification in Fig. 6f. **m,** Control experiment showing specificity of the assay, as fluorescent signal is not detected when cell lysate was pre-treated with 0.5 μM MG132 prior to addition of fluorogenic peptide substrate, n=3; one-way ANOVA: P < 0.0001 with post hoc Tukey test. Bar graphs data presented as normalized mean ± SEM. *P < 0.05, **P < 0.001, ***P < 0.0001; scale bar = 10 μm; WT, wild-type; ns, not significant. Source data are provided as a Source Data file.

Supplementary Fig. 6.

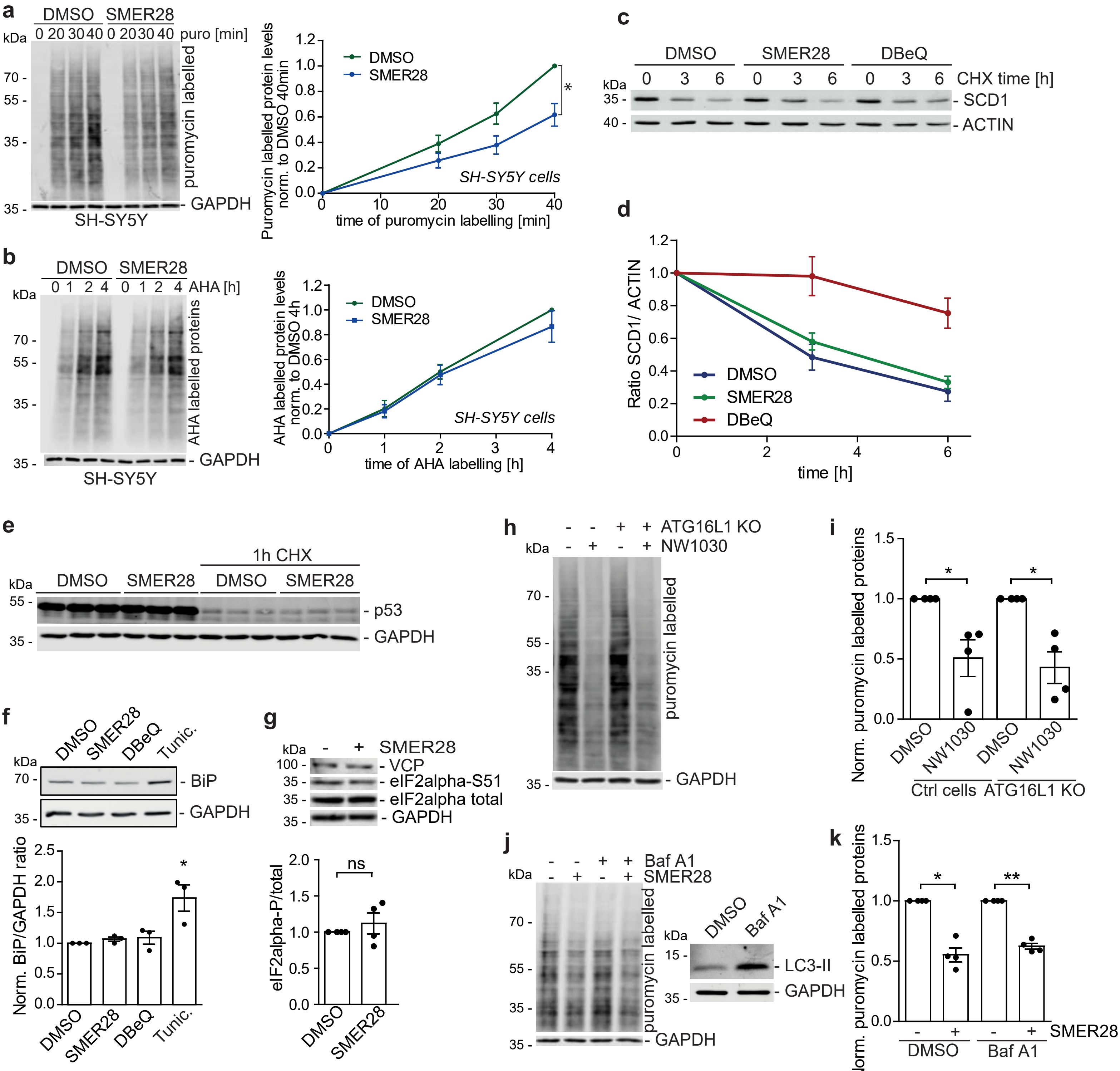

Supplementary Fig.6. SMER28 increases degradation of misfolded, but not healthy proteins.

a, SH-SY5Y cells were pre-treated with 20 µM SMER28 or DMSO for 4 h, followed by treatment with puromycin for indicated time points, n=3, P = 0.0215. **b,** SH-SY5Y cells were pre-treated for 1 h with 20 µM SMER28 or DMSO in medium deprived of methionine, followed by addition of methionine analog L-azidohomoalanine (AHA) for indicated time points. Cells were lysed and analysed by Western blotting using streptavidin antibody, n=3. **c, d,** Rate of SCD1 degradation is not influenced by SMER28 treatment. Cells were co-treated with Cycloheximide (CHX, 50 µg/ml) for the indicated time points, n=3; quantification in (**d**). **e,** Rate of p53 degradation is not affected by SMER28 treatment. HeLa cells were pre-treated with 20 µM SMER28 for 4 h, followed by treatment with Cycloheximide (CHX, 50 µg/ml) for 1 h. **f,** HeLa cells were treated with 20 µM SMER28, 10 µM DBeQ or 2.5 µM Tunicamycin for 8 h, n=3, and UPR induction measured by analysing BiP levels; one-way ANOVA: P = 0.0082 with post hoc Tukey test, Tunicamycin P = 0.011. **g,** HeLa cells were treated with 20 µM SMER28 for 6 h, n=4, and UPR induction measured by analysing phosphorylation of eIF2α, n=4; one sample t test. **h, i,** ATG16L1 knockout and control cells were pre-treated with 10 µM NW1030 or DMSO for 4 h, followed by treatment with puromycin for 15 min, quantification in (**i**), n=4; one sample t test, control P = 0.0483, ATG16KO P = 0.0227. **j, k,** HeLa cells were treated with 20 µM SMER28 or DMSO with or without 400 nM BafA1 for 4 h prior to treatment with puromycin for 15 min. Control blot showing accumulation of LC3-II upon BafA1 treatment, quantification in (**k**), n=4, DMSO P = 0.0046, BafA1 P = 0.0006. Bar graphs data presented as normalized mean ± SEM. *P < 0.05, **P < 0.001, ***P < 0.0001; ns, not significant. Source data are provided as a Source Data file.

Supplementary Fig. 7.

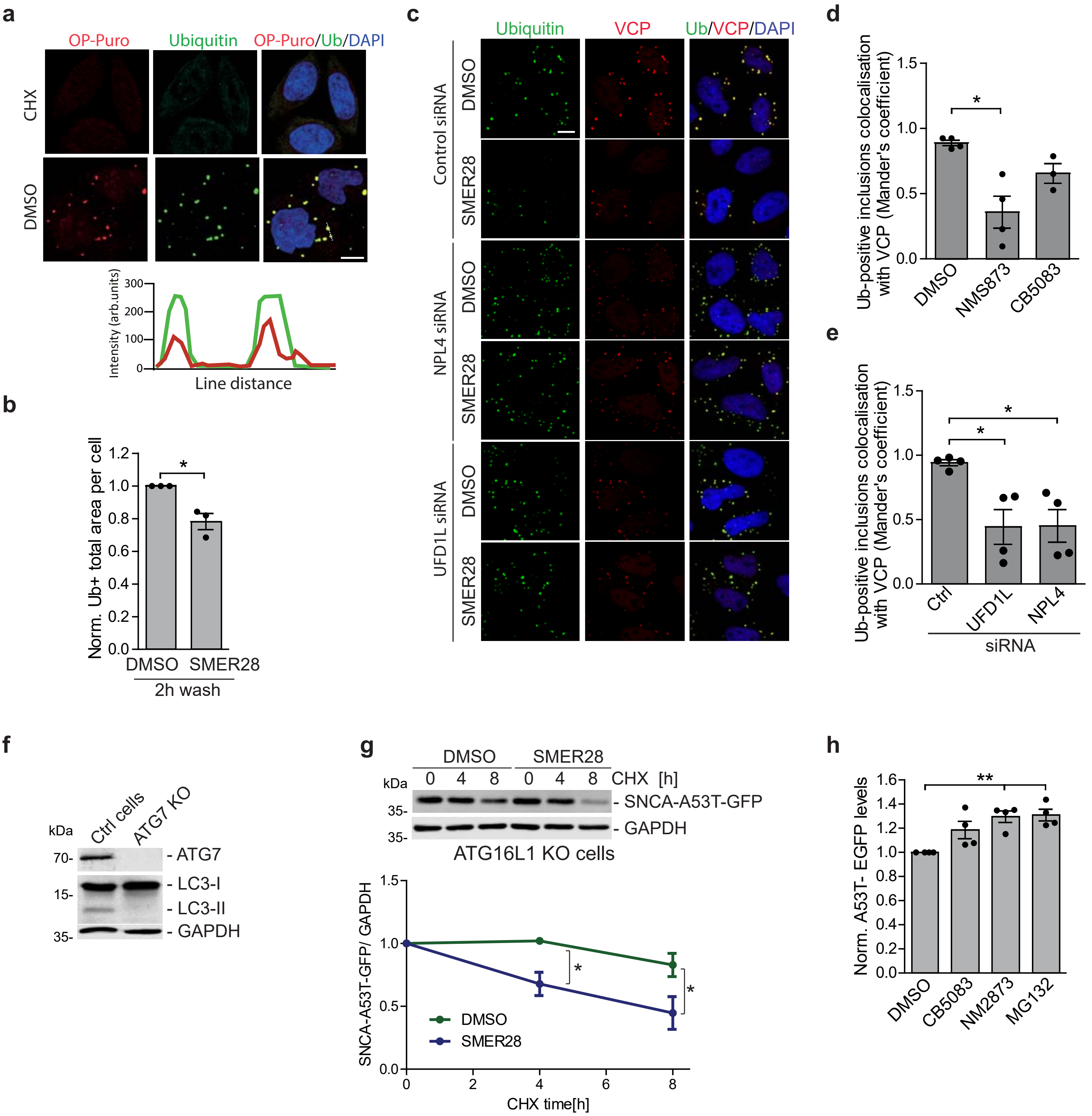

Supplementary Fig. 7. SMER28 prevents accumulation of misfolded proteins inclusions and increases clearance of aggregate-prone α-synuclein.

a, Control experiment. HeLa cells were treated with O-propargyl-puromycin (OP-Puro) with or without Cycloheximide (CHX, 50 µg/ml) for 4 h. Cells were fixed and OP-Puro and ubiquitin-positive structures were visualized. Line scans indicate the degree of colocalization between OP-Puro (red) and Ubiquitin (green) in lines drawn within the magnified images. The intensity profiles are presented as arbitrary units (arb. units). **b,** HeLa cells were treated with puromycin for 3 h, followed by washout and chase for 2 h in presence of DMSO or 20 µM SMER28. Cells were fixed, stained for ubiquitin-positive structures and total area of Ub+ per cell was quantified, n=3; SMER28 treated cells were normalized to DMSO in washout conditions; one sample t test. **c, e,** HeLa cells were treated with siRNA against UFD1L or NPL4 and after 48 h were pre-treated with 20 µM SMER28 or DMSO for 1 h followed by treatment with puromycin for 4 h, representative images in (**c**) and Manders' Colocalisation Coefficient analysis in (**e;** one-way ANOVA: $P = 0.0146$ with post hoc Tukey test, UFD1L $P = 0.0238$, NPL4 $P = 0.0258$), statistical analysis in Fig. 8c, n=4. **d,** Quantification of data from Fig. 8a. HeLa cells were pre-treated with 10 µM NMS873 or 5 µM CB-5083 for 1 h, followed by addition of puromycin for 4 h. Cells were fixed and stained for VCP puncta and ubiquitin-positive structures, Manders' Colocalisation Coefficient analysis, n=4; one-way ANOVA: $P = 0.0061$ with post hoc Tukey test, NMS873 $P = 0.0048$. **f,** HeLa ATG7 knock-out cells validation. Cells were lysed and analysed for ATG7 and LC3-II levels. **g,** ATG16L1 knockout HeLa cells were treated with Cycloheximide (CHX, 50 µg/ml) in presence of DMSO or 20 µM SMER28 for indicated time points, n=5; paired two-tailed Student's t test. **h,** ATG7 knockout HeLa cells stably expressing A53T-SNCA-EGFP were treated with 0.5 µM CB-5083, 1 µM NMS873 or 1 µM MG132 alone for 24 h, followed by FACS analysis; one-way ANOVA: $P = 0.0031$ with post hoc Tukey test. Bar graphs data presented as normalized mean ± SEM. *$P < 0.05$, **$P < 0.001$, ***$P < 0.0001$; scale bar = 10 µm; ns, not significant. Source data are provided as a Source Data file.

Supplementary Fig. 8. FACS gating strategies for HeLa cells stably expressing A53T-SNCA-EGFP, Ub-G76V-GFP or SRAI-LC3B reporter

For analysis by flow cytometry, HeLa cells stably expressing A53T-SNCA-EGFP, Ub-G76V-GFP or SRAI-LC3B reporter were isolated using forward scatter (FSC) and side scatter (SSC) properties. HeLa cells were distinguished from debris (lower FSC and SSC) and clumps (higher FSC and SSC). To ensure the assessed population consisted of single cells, single cells were differentiated from doublets by comparing the area of forward scatter (FSC-A) to the height of forward scatter (FSC-H). Cells in which FSC-H is disproportionately high compared to FSC-A are likely to be doublets and were therefore excluded from analysis. For A53T-SNCA-EGFP and Ub-G76V-GFP were defined by thresholding against unstained, wild-type HeLa. For SRAI-LC3B cells to isolate cells with sufficient expression of the SRAI-LC3B reporter protein, TOLLES-positive HeLa were defined by thresholding against unstained, wild-type HeLa. Cells with greater VL2-A fluorescence than the distribution of unstained HeLa cells (TOLLES-CFP-A+) were selected for further analysis. To obtain a quantitative ratiometric readout, we derived the median TOLLES:YPet ratio of the TOLLES-CFP-A+ population. The TOLLES:YPet ratio was defined using the “Derive Parameters” function as (VL2-A*100)/BL1-A.

Supplementary Fig. 8.

SEQUENTIAL GATING STRATEGY FOR ANALYSIS OF ALPHA-SYNUCLEIN EXPRESSING CELLS

1. HeLa cells population is defined by forward and side scatter properties

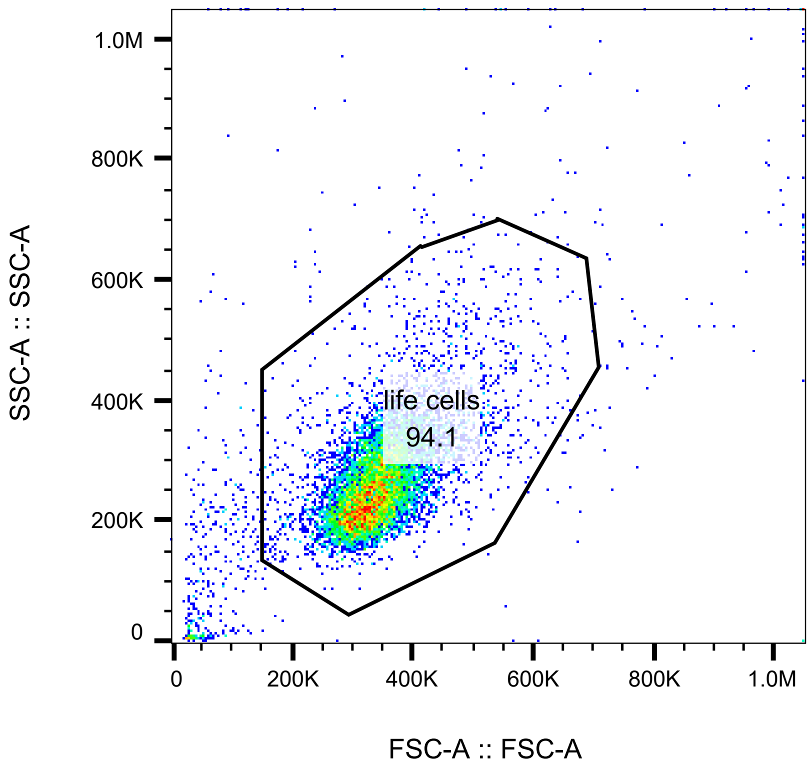

2. Single cells population is defined

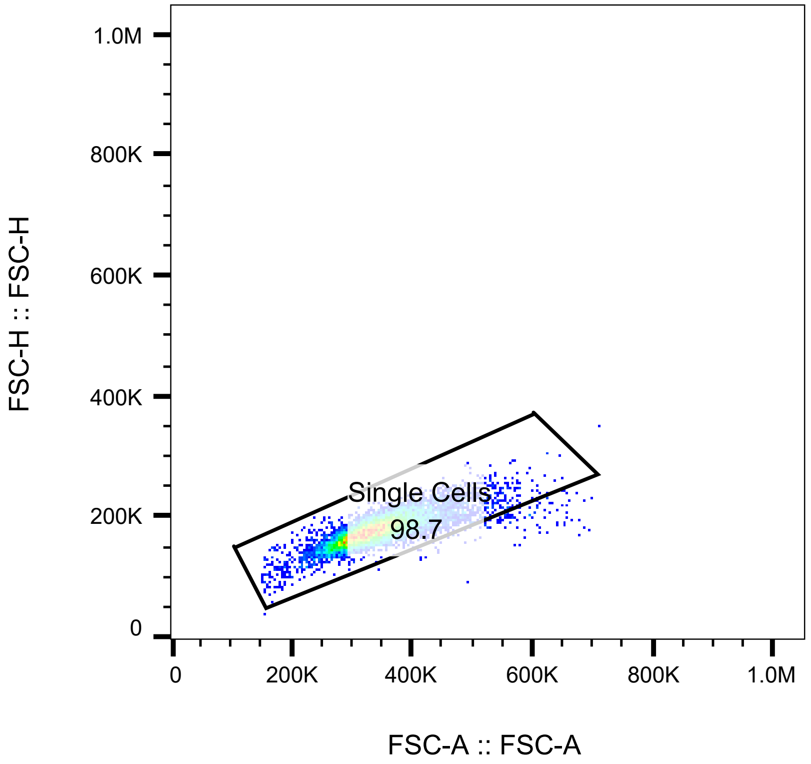

3. GFP positive cells population is defined using wild-type HeLa cells (not expressing GFP). Median value of GFP positive population is calculated.

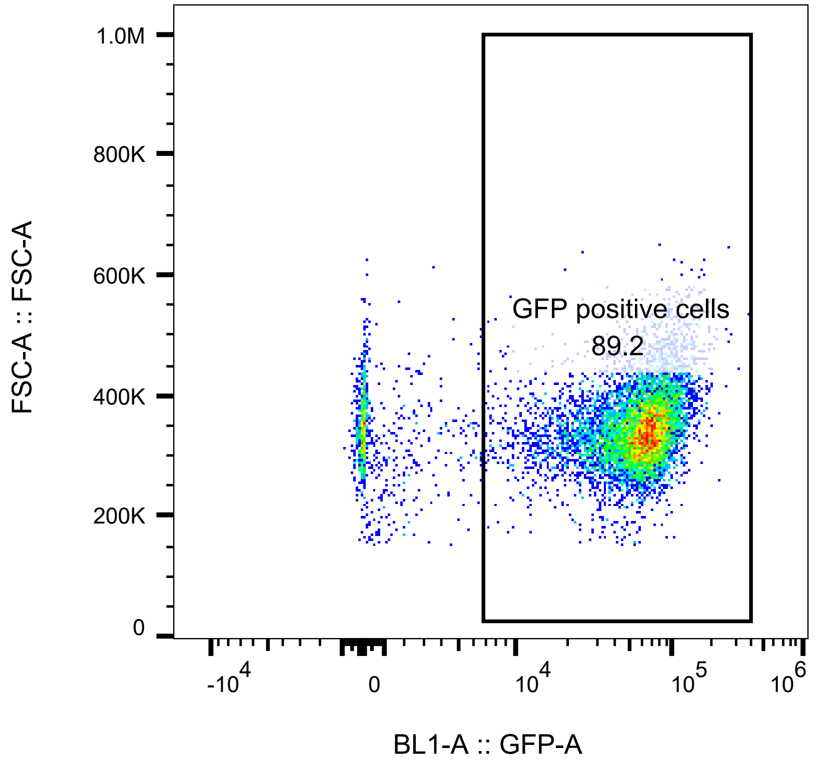

Supplementary Fig. 8 - continued.

SEQUENTIAL GATING STRATEGY FOR ANALYSIS OF UB-G76V-GFP DEGRON LEVELS

1. HeLa cells population is defined by forward and side scatter properties

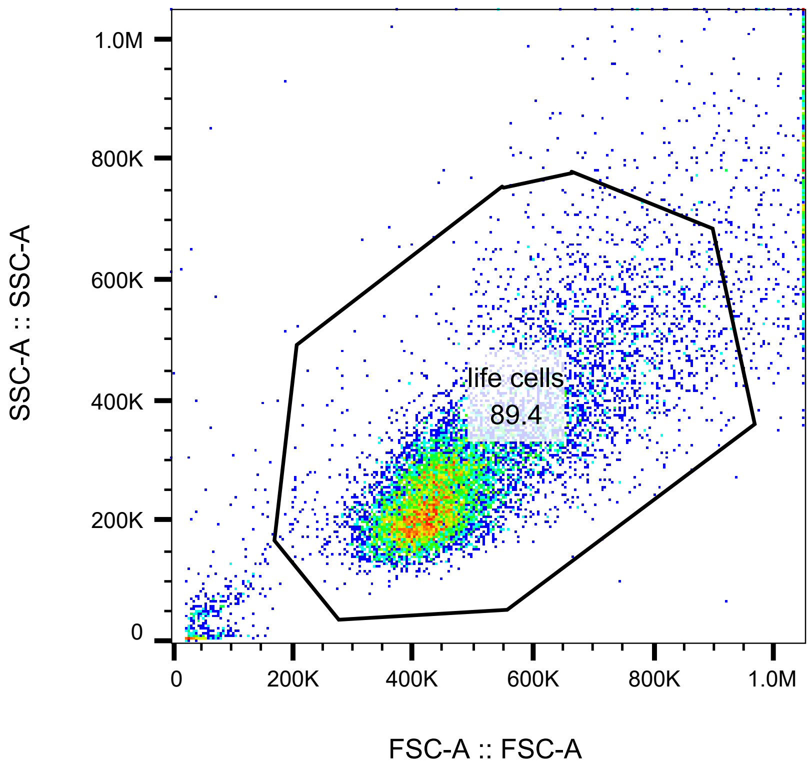

2. Single cells population is defined

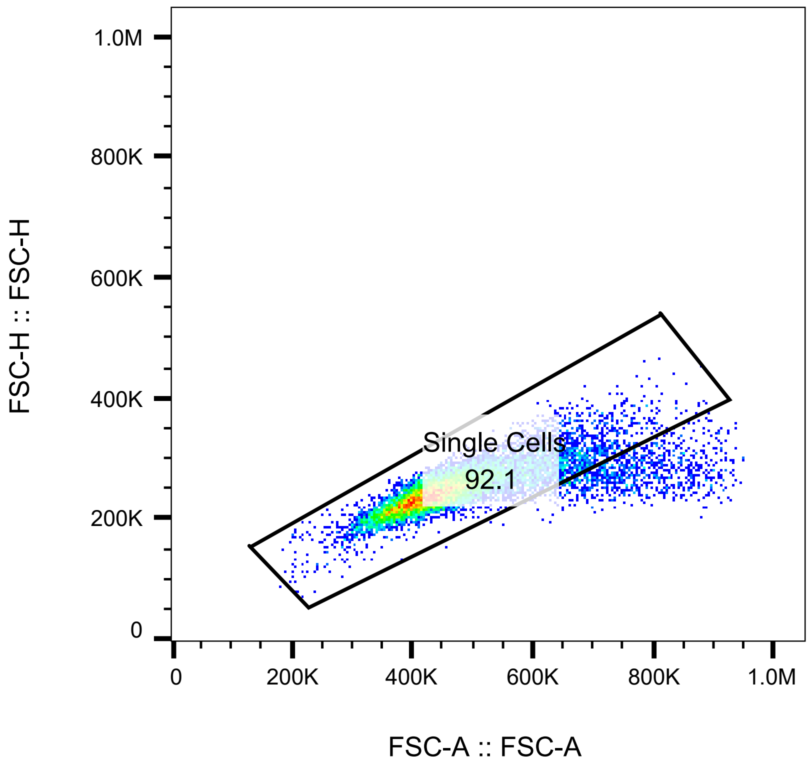

3. GFP positive cells population is defined using wild-type HeLa cells (not expressing GFP). Percentage of GFP positive cells is assessed.

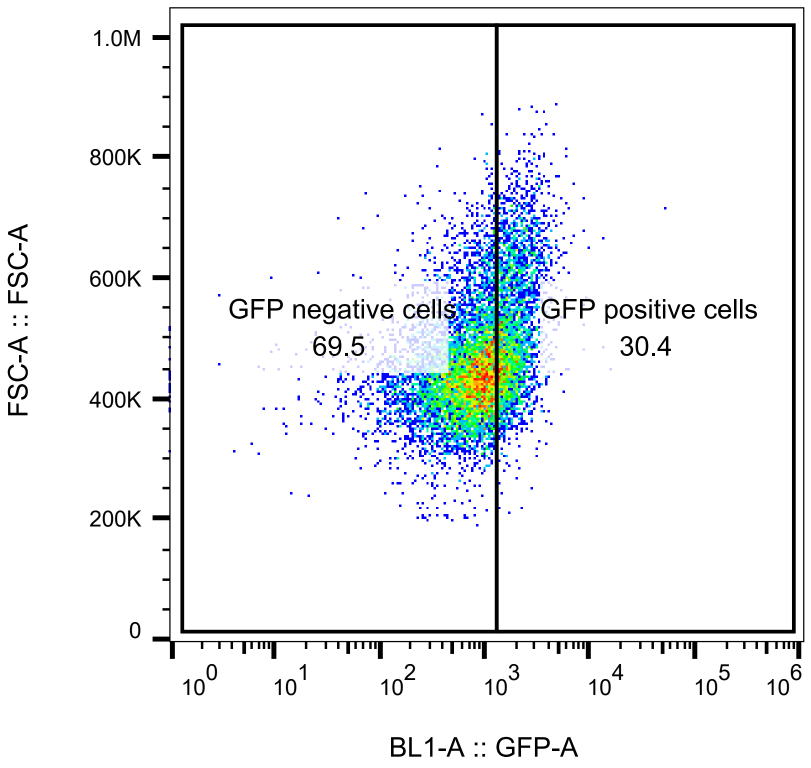

Supplementary Fig. 8 – continued.

SEQUENTIAL GATING STRATEGY FOR ANALYSIS OF SRAI-LC3B EXPRESSING CELLS

1. HeLa cell population defined using forward scatter (FSC) and side-scatter (SSC) properties

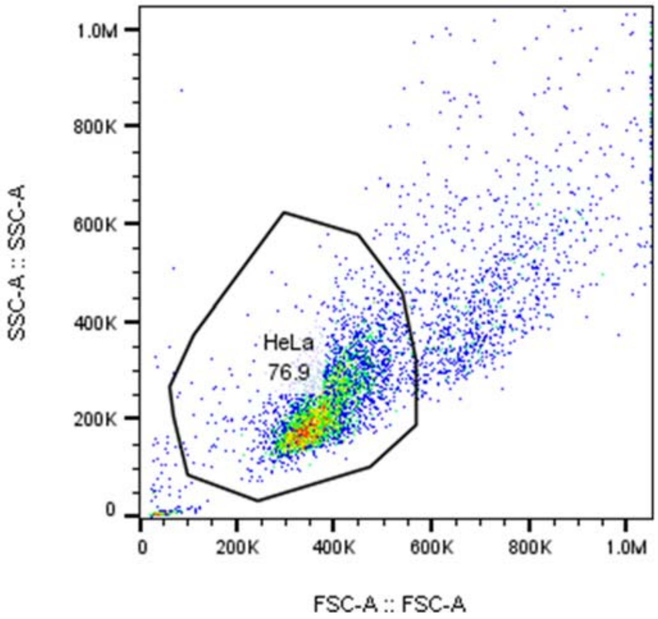

2. Single cell population is defined by comparison of FSC-A to FSC-H

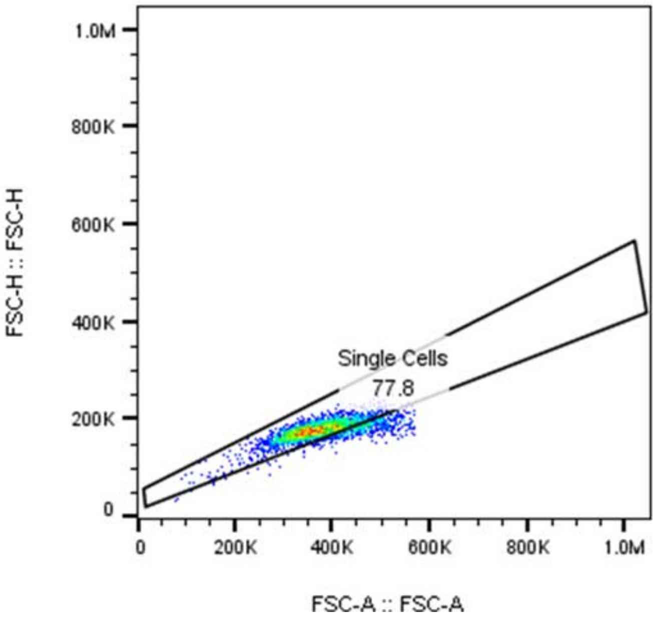

3. TOLLES-positive HeLa were defined by thresholding against unstained, wild-type HeLa. Cells expressing TOLLES (TOLLES-CFP-A+) were subsequently assessed. The measure used was the median TOLLES:YPet ratio of the TOLLES-CFP-A+ population. The TOLLES:YPet ratio was defined using the “Derive Parameters” function as (VL2-A*100)/BL1-A.

Unstained HeLa

SRAI-LC3B-expressing HeLa

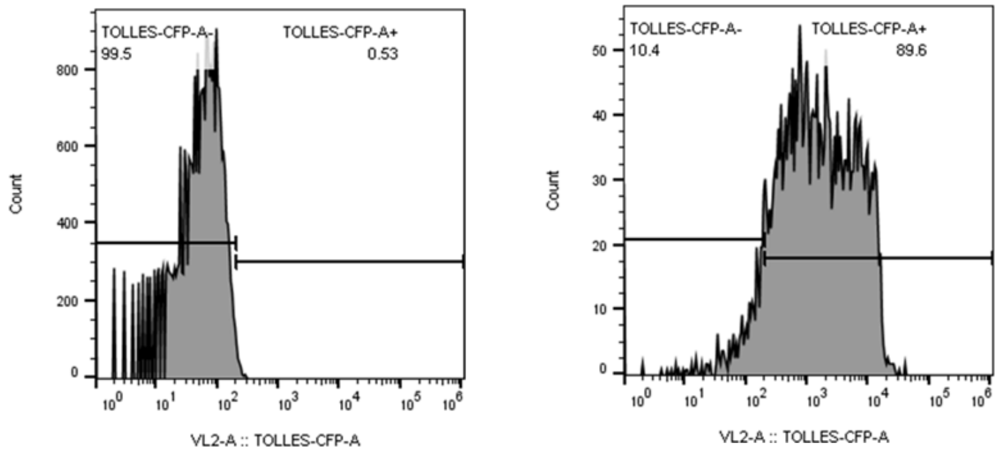

Supplementary Fig. 9. Uncropped Western blots

Supp. Fig 1d Mutant HTT levels in patient-derived fibroblasts

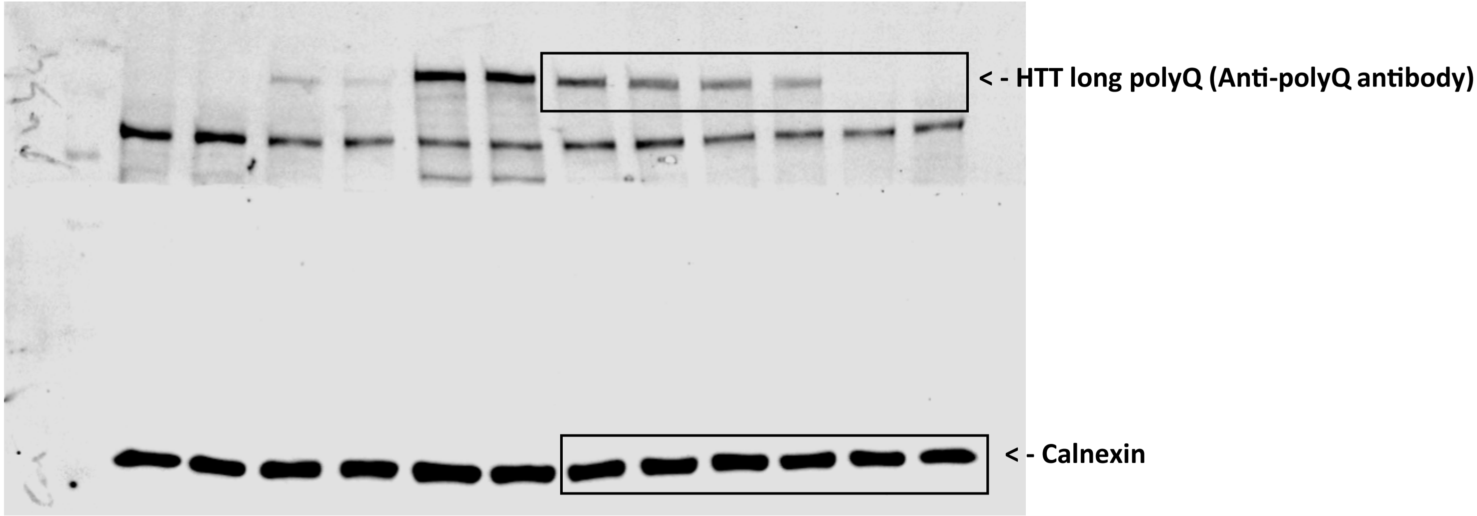

Supp.Fig 2b DARTS assay upon SMER28 and analogs treatments

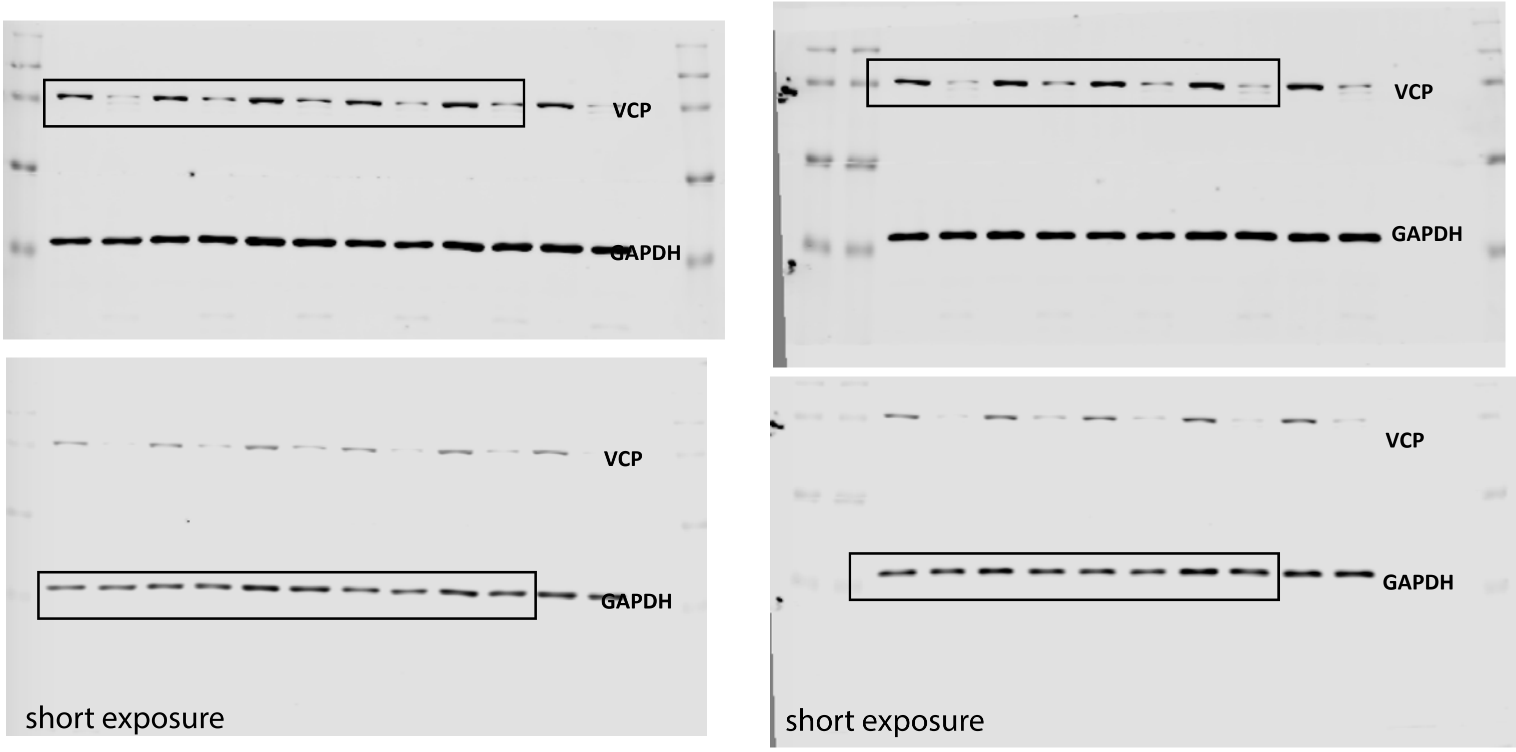

Supplementary Fig. 9. Uncropped Western blots - continued

Supp.Fig 3e LC3-II levels in SMER28 +-DBeQ

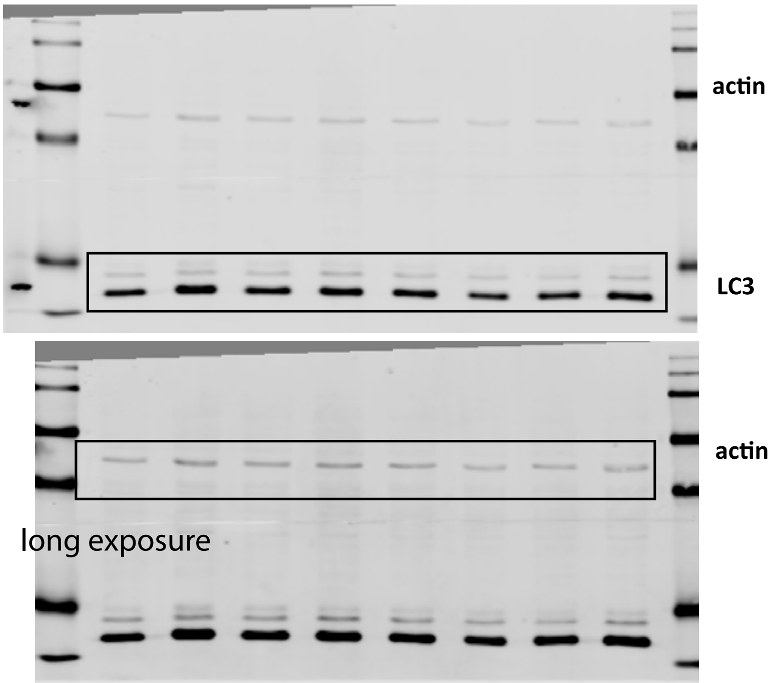

Supp.Fig 3e LC3-II levels in SMER28 +CB-5083 and NMS873

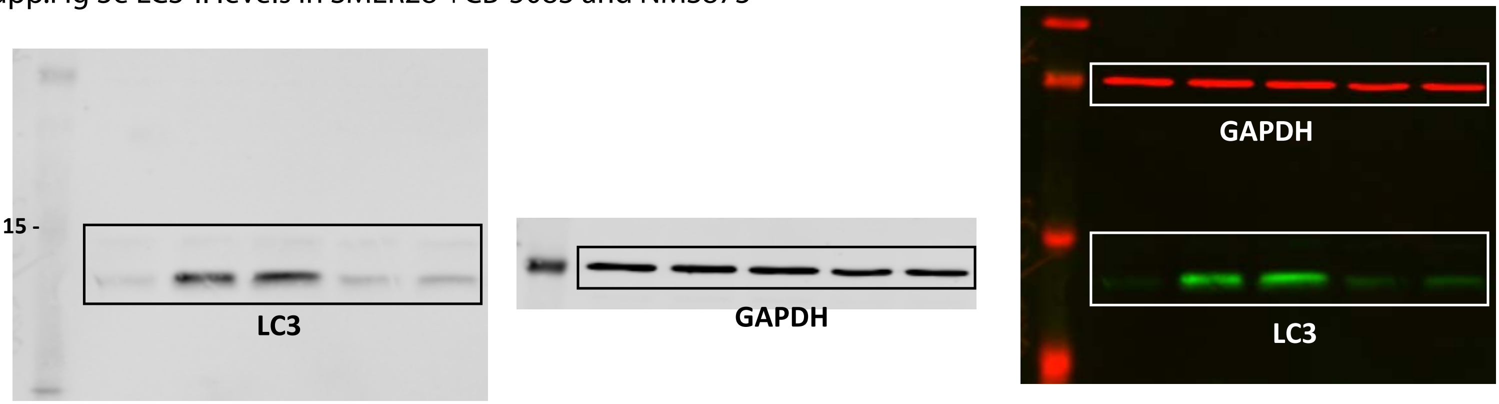

Supp.Fig 3g TEX264 and p62 levels upon EBSS and SMER28

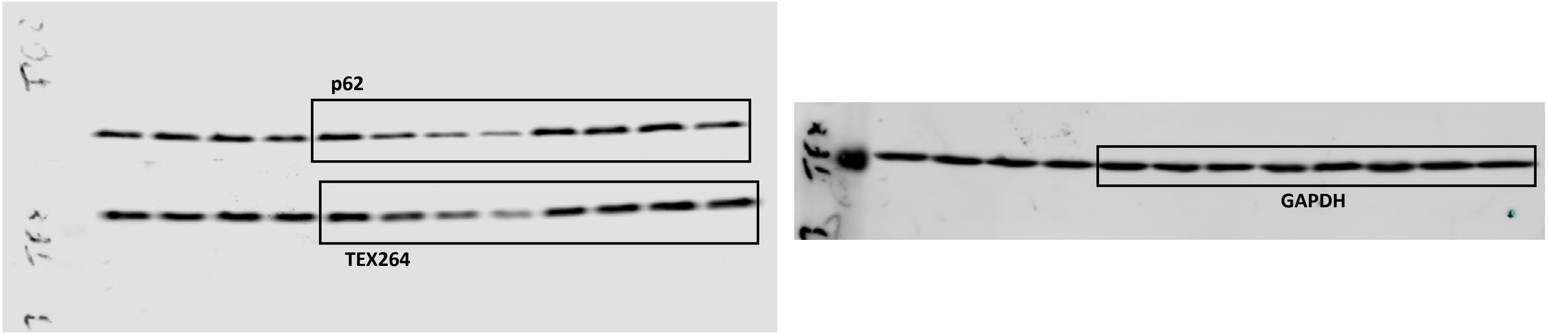

Supp.Fig 3h AKT phosphorylation levels upon SMER28

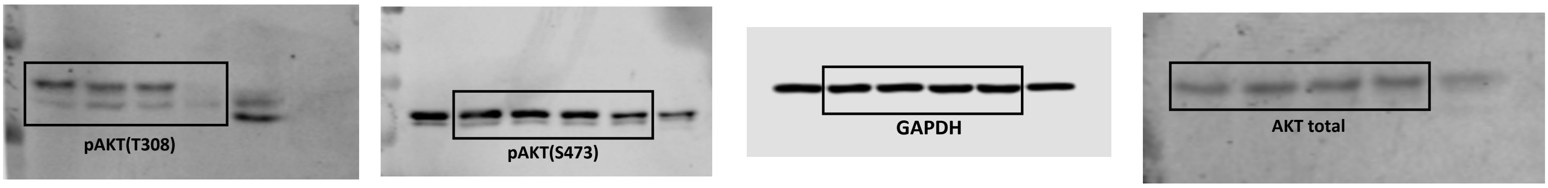

Supplementary Fig. 9. Uncropped Western blots - continued

Supp. Fig 4c HTTQ7 and HTTQ111 levels upon NW1030 treatment

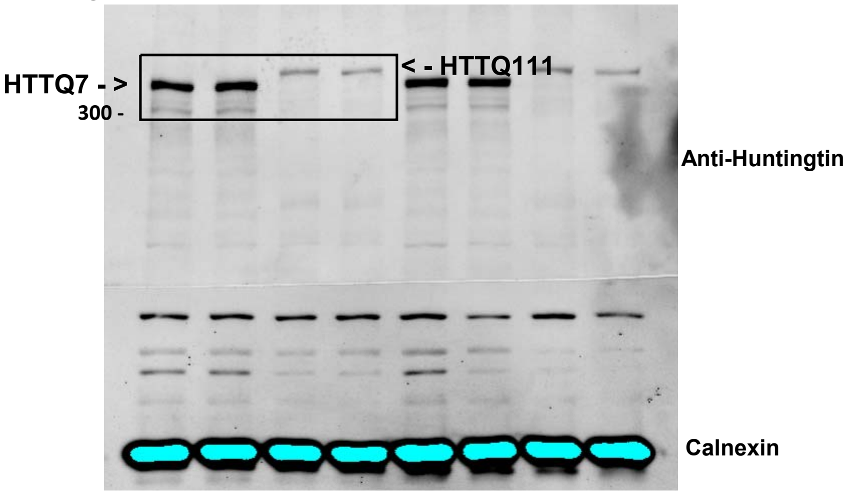

lower exposure for Calnexin

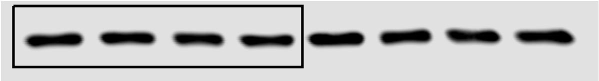

Supp. Fig 4d LC3 levels upon NW1030 with VCP inhibitors

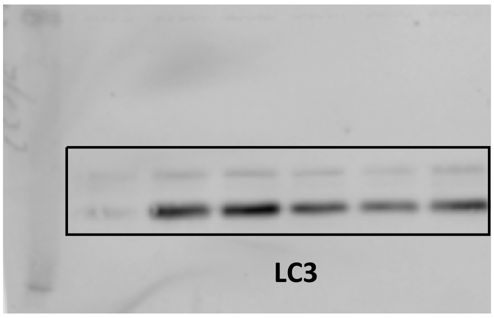

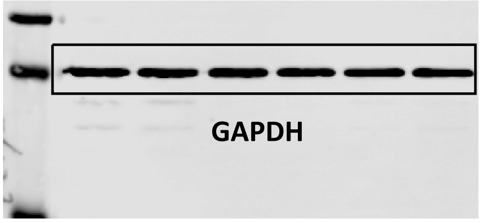

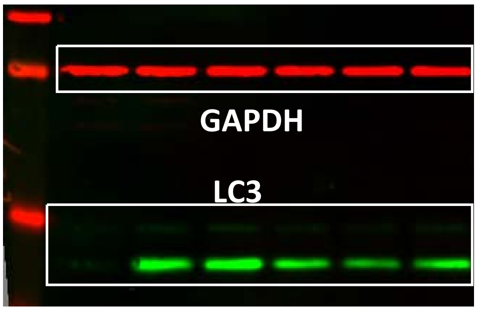

Supplementary Fig. 9. Uncropped Western blots - continued

Supp.Fig 5b PI3K IP upon SMER28 and DBeQ treatment

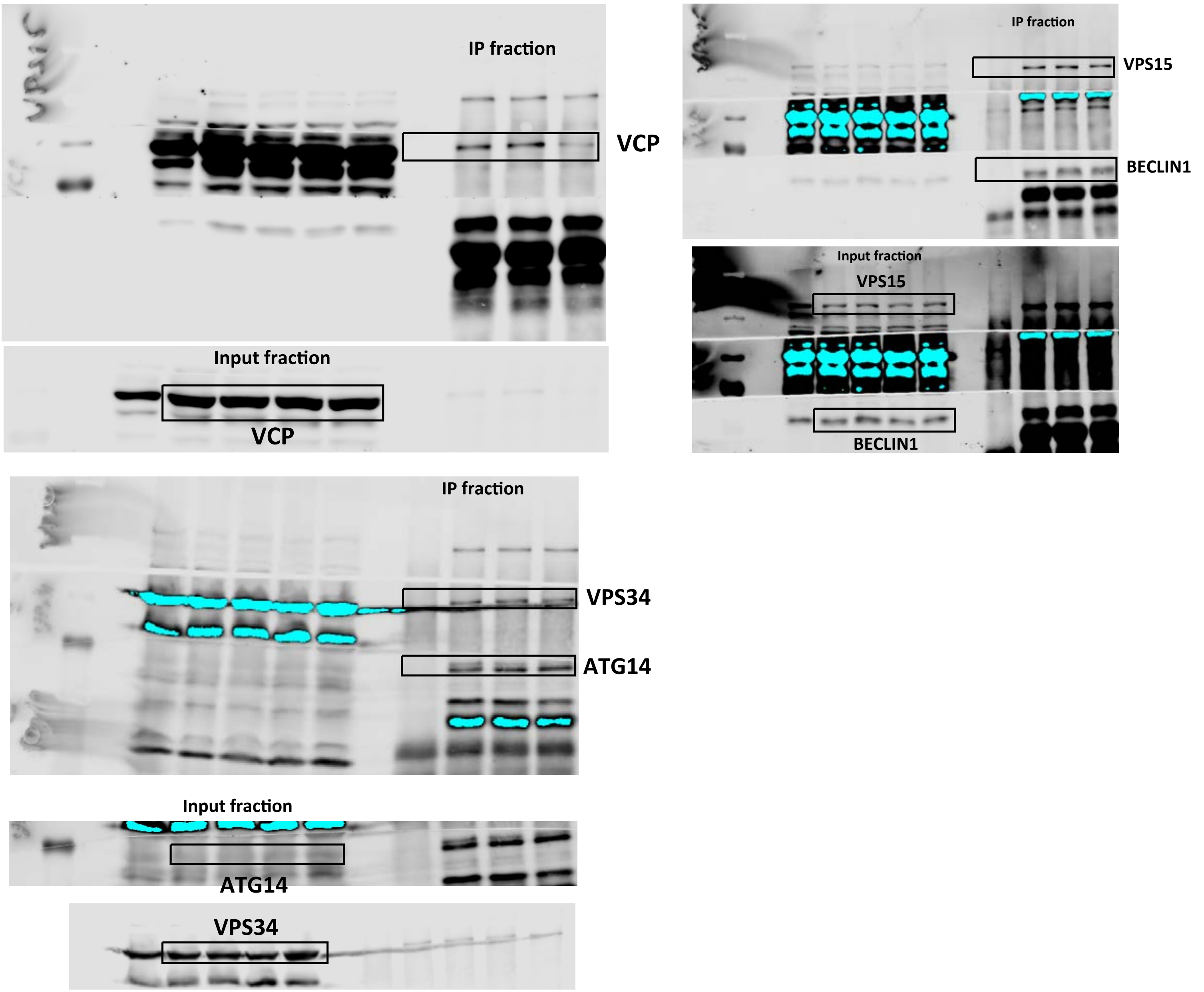

Supplementary Fig. 9. Uncropped Western blots - continued

Supp. Fig 5d in vitro binding to VCP-GST beads upon SMER28

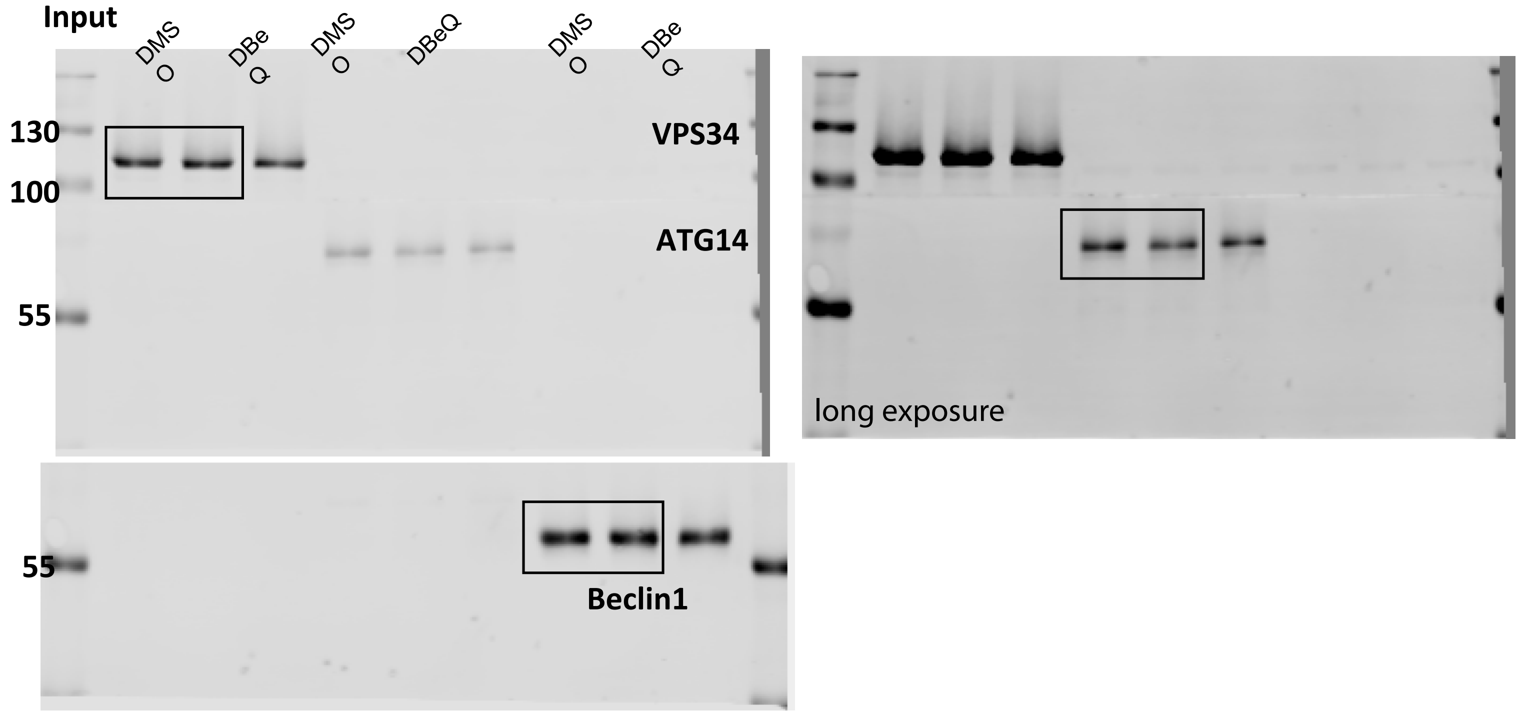

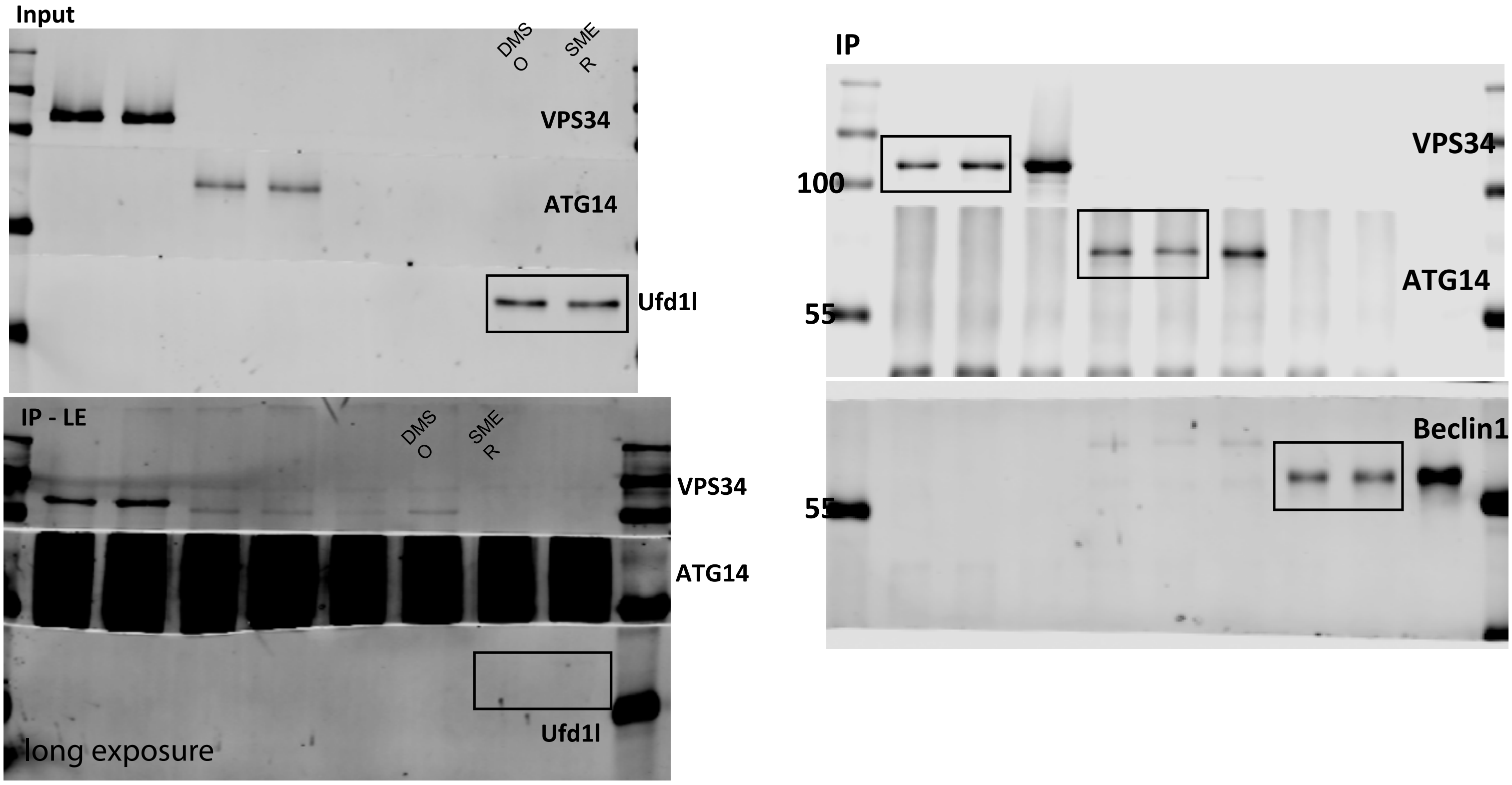

Supp.Fig 5g VCP knockdown efficiency

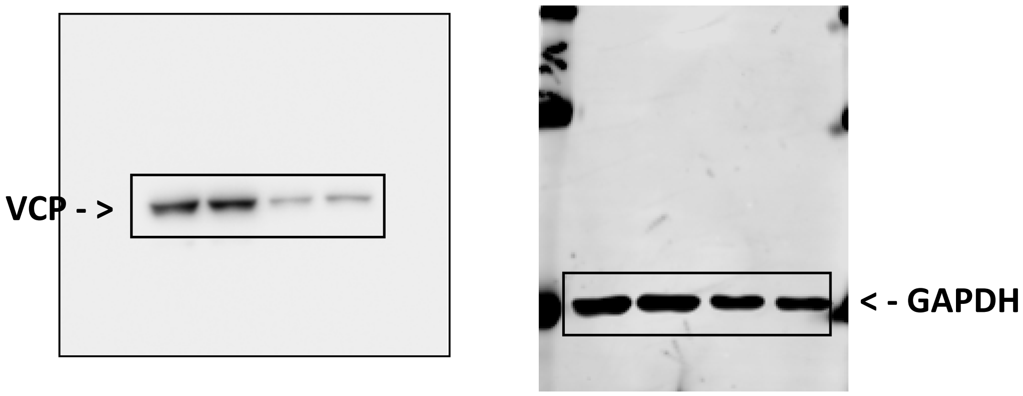

Supplementary Fig. 9. Uncropped Western blots - continued

Supp.Fig 5i UFD1L and NPL4 knockdown efficiency

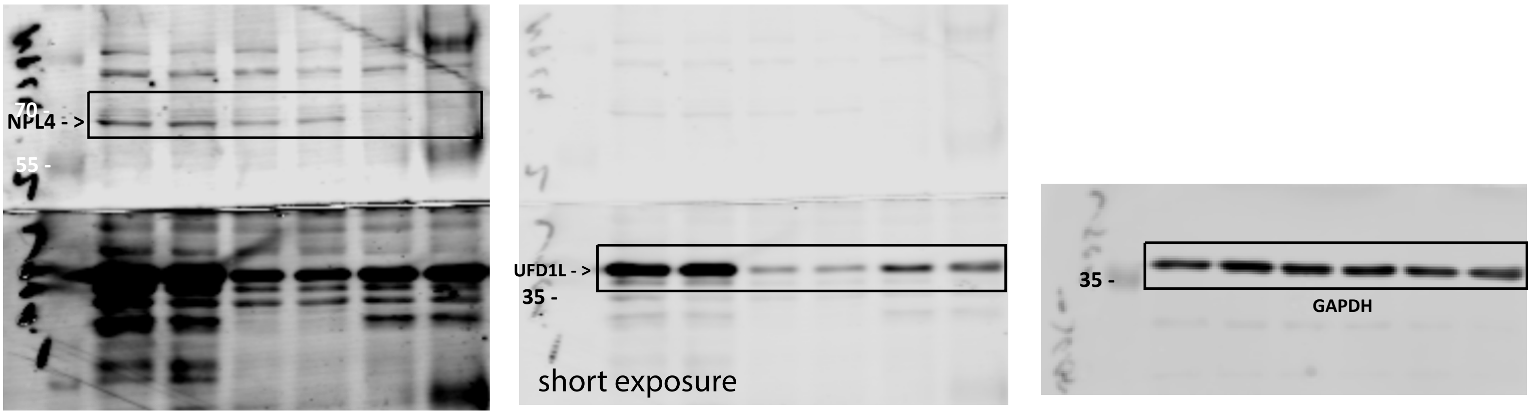

Supp.Fig 5j UFD1L and NPL4 interaction with VCP upon SMER28 treatment - in vitro binding assay

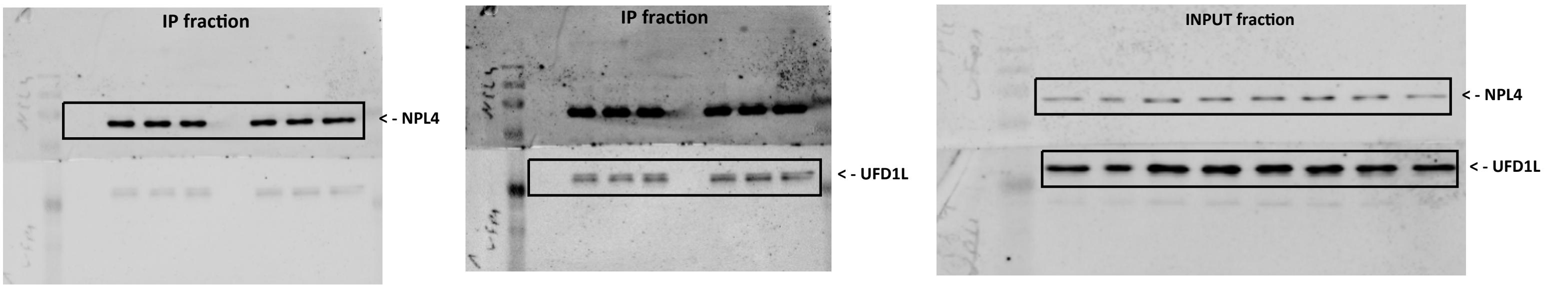

Supp.Fig 5k UFD1L and NPL4 interaction with VCP upon SMER28 treatment

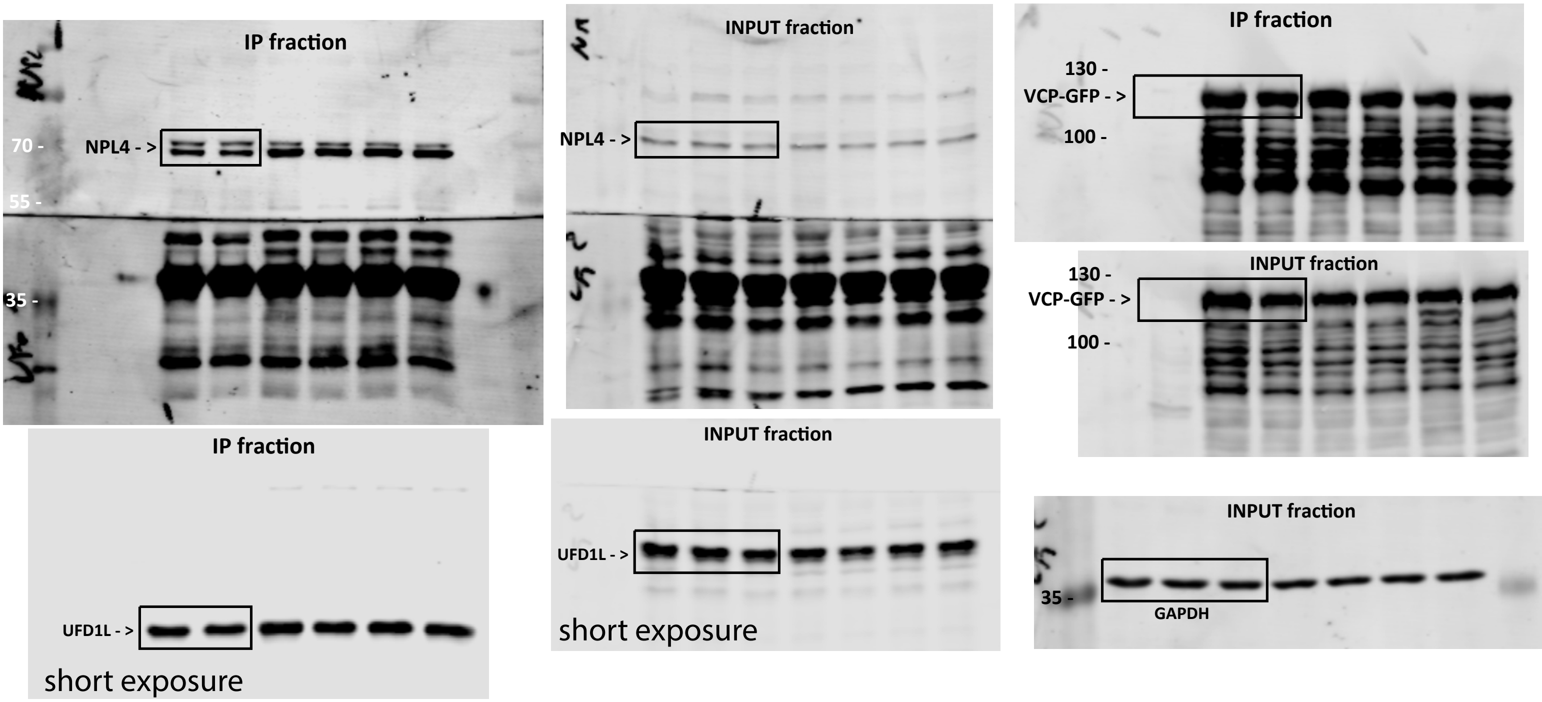

Supplementary Fig. 9. Uncropped Western blots - continued

Supp.Fig 6a Puromycin-labelled proteins upon SMER28 treatment in SH-SY5Y cells

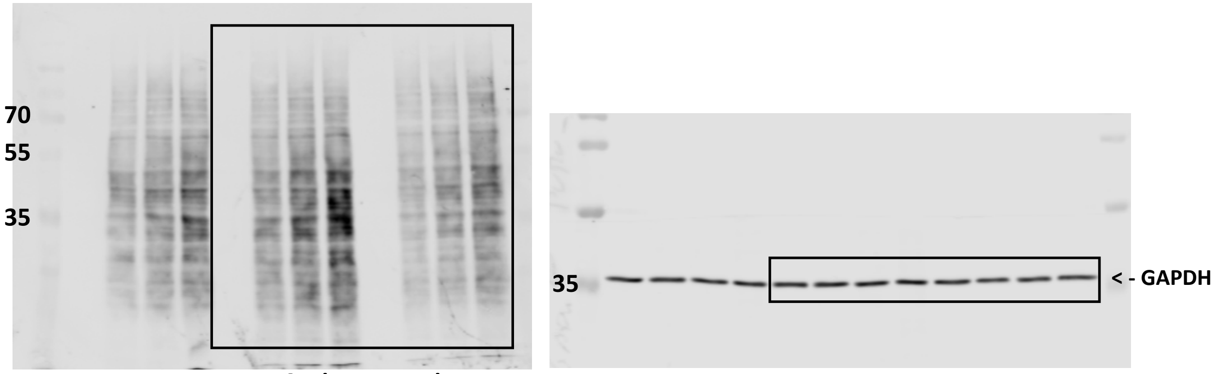

Anti-puromycin

Supp.Fig 6b AHA-labelled proteins upon SMER28 treatment in SH-SY5Y cells

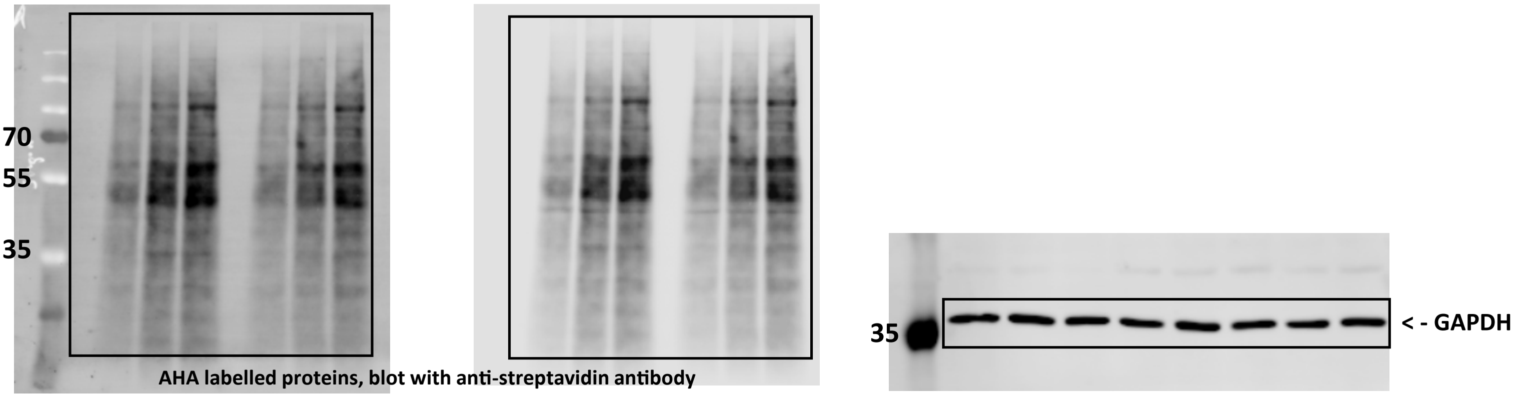

AHA labelled proteins, blot with anti-streptavidin antibody

Supp.Fig 6c and d. SCD1 (ERAD substrate) degradation upon SMER28 treatment

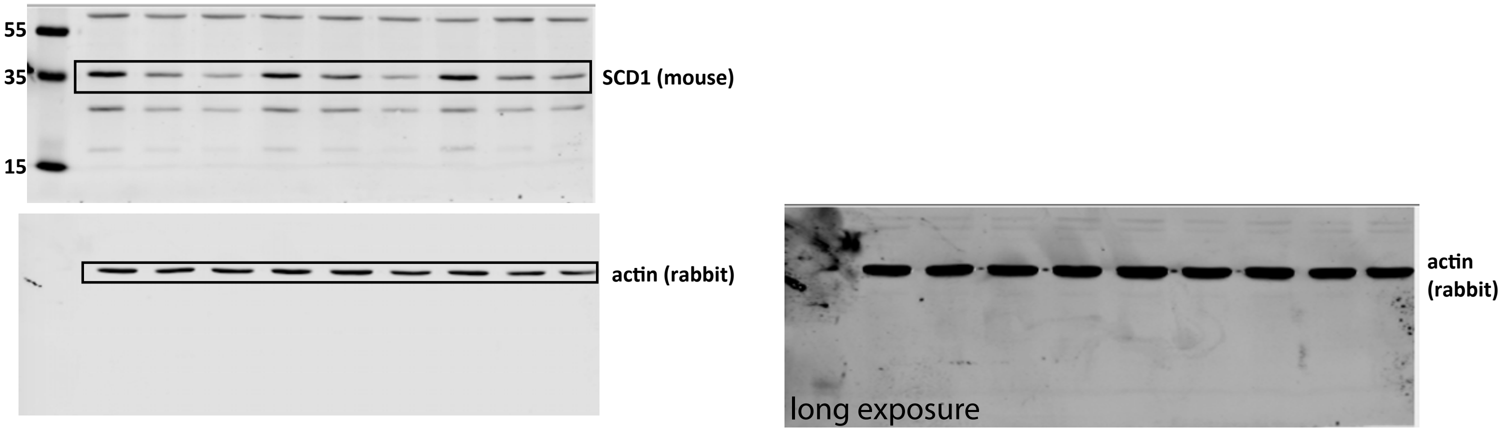

Supp.Fig 6e p53 degradation upon SMER28 treatment

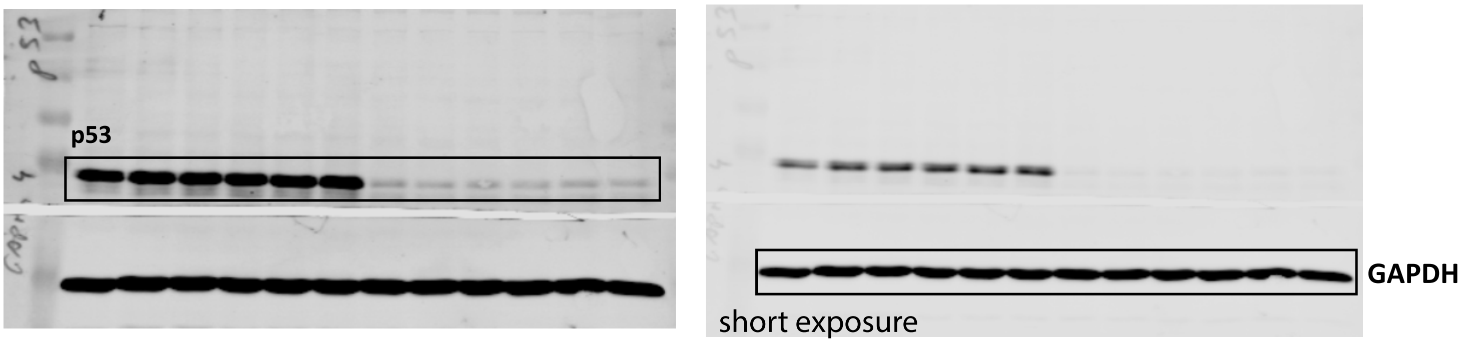

Supplementary Fig. 9. Uncropped Western blots - continued

Supp.Fig 6f BiP levels after SMER28 treatment

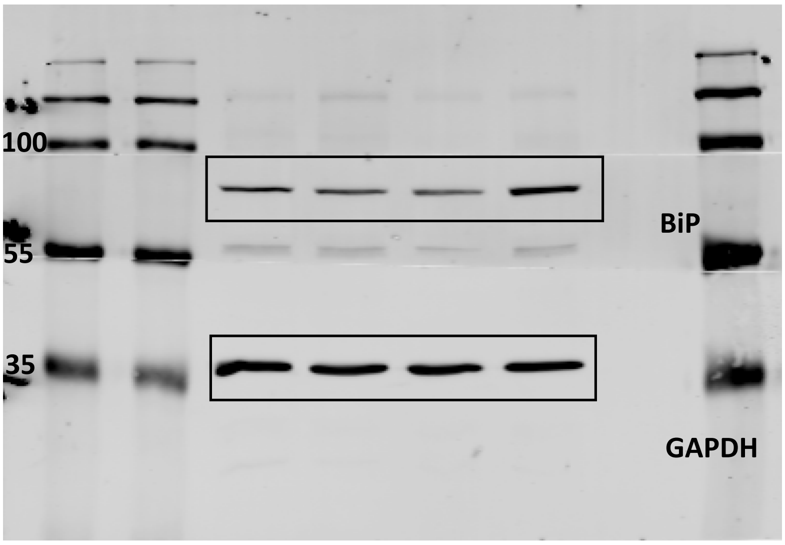

Supp.Fig 6g ER stress upon SMER28 treatment

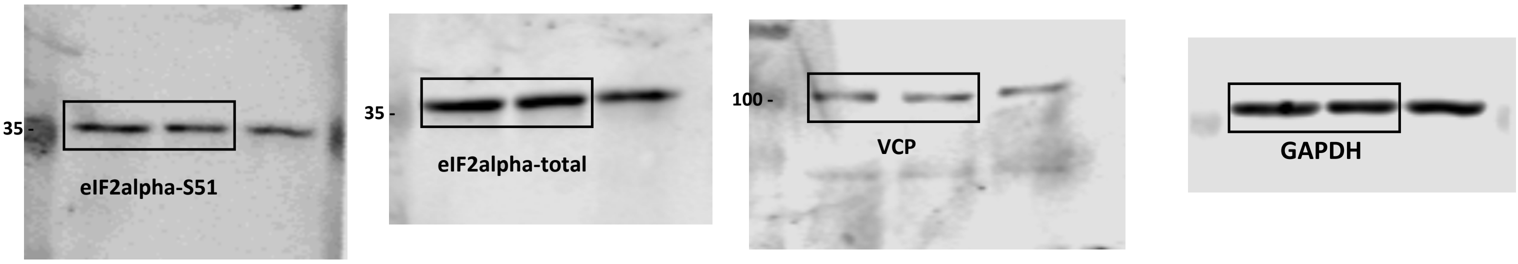

Supp.Fig 6h Puromycin-labelled proteins upon NW1030 treatment in ATG16 WT and ATG16 null HeLa cells

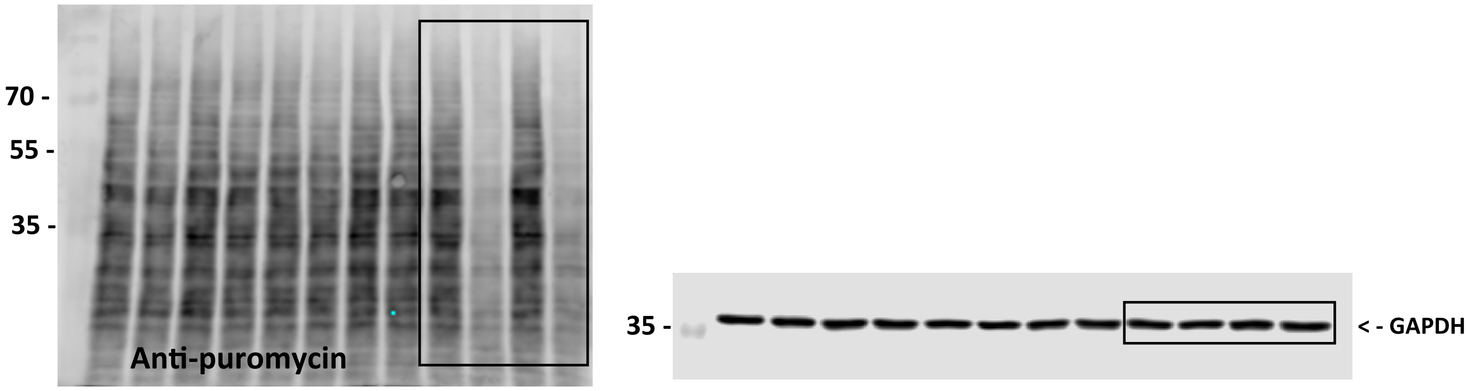

Supp.Fig 6j Puromycin-labelled proteins uponSMER28 treatment in Baf A1 conditions

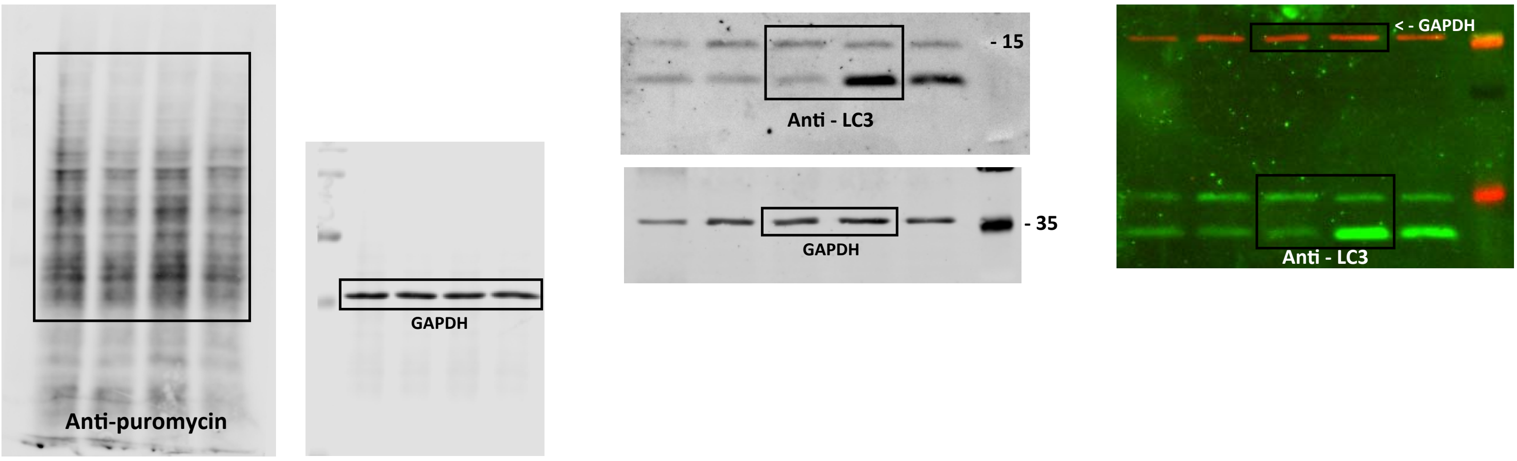

Supplementary Fig. 9. Uncropped Western blots - continued

Supp. Fig 7f ATG7 knockout cells - control blot

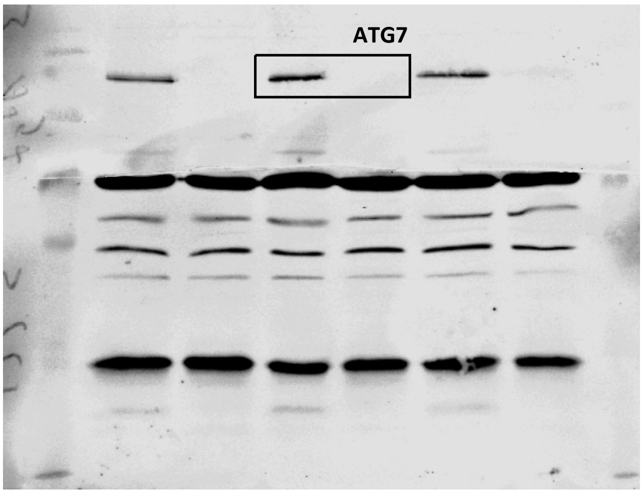

short exposure

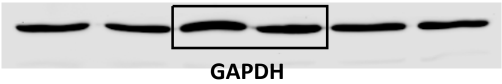

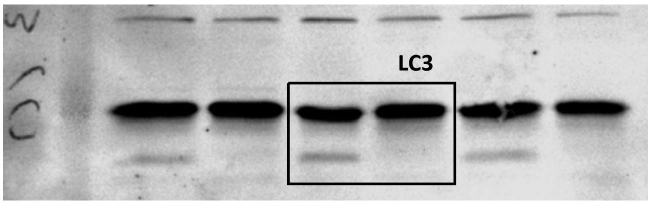

Supp.Fig 7g A53T-GFP levels upon SMER28 treatment in ATG16 null cells

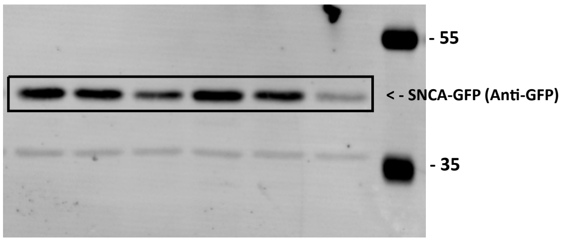

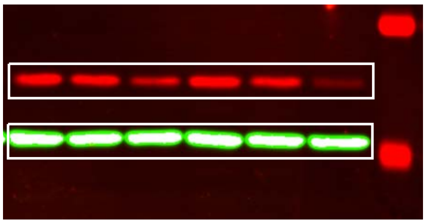

short exposure

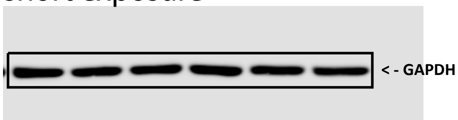

Supplement: Supplementary file 1 — Supplementary Information [file 41467_2022_31905_MOESM1_ESM.pdf]
