# Supplementary material for: Compounds activating VCP D1 ATPase enhance both autophagic and proteasomal neurotoxic protein clearance
Source: Nat Commun. 2022 Jul 16;13:4146. doi: 10.1038/s41467-022-31905-0 (PMC9288506; doi:10.1038/s41467-022-31905-0)
Supplement: Supplementary file 3 — Description of Additional Supplementary Files [file 41467_2022_31905_MOESM3_ESM.pdf]

File name: Supplementary Data 1

Description: Reverse competition assay identifies protein targets of SMER28.

File name: Supplementary Data 2

Description: The effect of SMER28 tested at a concentration of 10 µM against a panel of 123 kinases.

File name: Supplementary Data 3

Description: The effect of SMER28 structural analogs on kinase activity.
