# Supplementary material for: Compounds activating VCP D1 ATPase enhance both autophagic and proteasomal neurotoxic protein clearance
Source: Nat Commun. 2022 Jul 16;13:4146. doi: 10.1038/s41467-022-31905-0 (PMC9288506; doi:10.1038/s41467-022-31905-0)
Supplement: Supplementary file 7 — Reporting Summary [file 41467_2022_31905_MOESM7_ESM.pdf]

nature portfolio

Corresponding author(s): David C Rubinsztein

Last updated by author(s): 13/06/2022

Reporting Summary

Nature Portfolio wishes to improve the reproducibility of the work that we publish. This form provides structure for consistency and transparency in reporting. For further information on Nature Portfolio policies, see our Editorial Policies and the Editorial Policy Checklist.

Statistics

For all statistical analyses, confirm that the following items are present in the figure legend, table legend, main text, or Methods section.

n/a	Confirmed	
☐	☒	The exact sample size (*n*) for each experimental group/condition, given as a discrete number and unit of measurement
☐	☒	A statement on whether measurements were taken from distinct samples or whether the same sample was measured repeatedly
☐	☒	The statistical test(s) used AND whether they are one- or two-sided<br>*Only common tests should be described solely by name; describe more complex techniques in the Methods section.*
☒	☐	A description of all covariates tested
☒	☐	A description of any assumptions or corrections, such as tests of normality and adjustment for multiple comparisons
☐	☒	A full description of the statistical parameters including central tendency (e.g. means) or other basic estimates (e.g. regression coefficient) AND variation (e.g. standard deviation) or associated estimates of uncertainty (e.g. confidence intervals)
☐	☒	For null hypothesis testing, the test statistic (e.g. *F*, *t*, *r*) with confidence intervals, effect sizes, degrees of freedom and *P* value noted<br>*Give P values as exact values whenever suitable.*
☒	☐	For Bayesian analysis, information on the choice of priors and Markov chain Monte Carlo settings
☒	☐	For hierarchical and complex designs, identification of the appropriate level for tests and full reporting of outcomes
☒	☐	Estimates of effect sizes (e.g. Cohen's *d*, Pearson's *r*), indicating how they were calculated

Our web collection on statistics for biologists contains articles on many of the points above.

Software and code

Policy information about availability of computer code

Data collection	no software was used for data collection
Data analysis	Excel 2016 (Microsoft office)<br>Prism v7 and v8 GraphPad<br>ZEN Black 2.6 Carl Zeiss Microscopy<br>Image Studio Lite LI-COR ver 5.2, Inc<br>ImageJ National Institute of Health, USA<br>Cellprofiler Broad Institute<br>FlowJo software FlowJo ver 10.7.1<br>Proteome Discoverer version 2.1.1.21 Thermofisher<br>TIBCO® Spotfire® Analyst 7.9.2 TIBCO<br>Spectronaut 14 Biognosys AG<br>The PyMOL Molecular Graphics System Schrödinger, Inc<br>Sequest HT

For manuscripts utilizing custom algorithms or software that are central to the research but not yet described in published literature, software must be made available to editors and reviewers. We strongly encourage code deposition in a community repository (e.g. GitHub). See the Nature Portfolio guidelines for submitting code & software for further information.

Data

Policy information about availability of data

All manuscripts must include a data availability statement. This statement should provide the following information, where applicable:

- Accession codes, unique identifiers, or web links for publicly available datasets
- A description of any restrictions on data availability
- For clinical datasets or third party data, please ensure that the statement adheres to our policy

Authors can confirm that all relevant data are included in the paper and/or its supplementary information files. All mass spectrometry proteomics data are available in the PRoteomics IDEntifications (PRIDE) database via ProteomeXchange. The Limited Proteolysis data used in this study are available in the PRIDE database under accession code PXD027750 [https://www.ebi.ac.uk/pride/archive/projects/PXD027750] (Username: reviewer_pxd027750@ebi.ac.uk, Password: zAl2GvVQ).The Chemoproteomic profiling of SMER28 data used in this study are available in the PRIDE database under accession code PXD034712 [https://www.ebi.ac.uk/pride/archive/projects/ PXD034712] (Username:reviewer_pxd034712@ebi.ac.uk, Password: D3Q9IZfS).
The custom R script used to compute dose response correlations was modified from the script available via GitHub repository [https://zenodo.org/record/6625705#.YqNQ19PMKUk]

Field-specific reporting

Please select the one below that is the best fit for your research. If you are not sure, read the appropriate sections before making your selection.

☒ Life sciences ☐ Behavioural & social sciences ☐ Ecological, evolutionary & environmental sciences

For a reference copy of the document with all sections, see nature.com/documents/nr-reporting-summary-flat.pdf

Life sciences study design

All studies must disclose on these points even when the disclosure is negative.

Sample size	The experiments assess parameters which are frequently tested in the lab (for example drug treatments to induce autophagy). Accordingly, the sample size was chosen based on previous experience with these experimental designs.
Data exclusions	No data was excluded from the analysis
Replication	Multiple replicates are reported for each experiment when possible. Each replicate was perform at the different time. Each replicate showed similar effect/result.
Randomization	Most experiments were done in single cell lines. Samples were allocated randomly into experimental groups
Blinding	Immunofluorescence analysis was blinded when possible. Western blot analysis was not blinded as it was not possible as the gel loading order needs to be defined.

Reporting for specific materials, systems and methods

We require information from authors about some types of materials, experimental systems and methods used in many studies. Here, indicate whether each material, system or method listed is relevant to your study. If you are not sure if a list item applies to your research, read the appropriate section before selecting a response.

Materials & experimental systems

n/a	Involved in the study	
☐	☒	Antibodies
☐	☒	Eukaryotic cell lines
☒	☐	Palaeontology and archaeology
☒	☐	Animals and other organisms
☒	☐	Human research participants
☒	☐	Clinical data
☒	☐	Dual use research of concern

Methods

n/a	Involved in the study	
☒	☐	ChIP-seq
☐	☒	Flow cytometry
☒	☐	MRI-based neuroimaging

Antibodies

Antibodies used	Primary antibodies: mouse anti-Flag M2 (Cat# F1804, RRID:AB_262044, 1:1000), and rabbit anti-Actin (Cat# A2066, RRID AB_476693; 1:1000) from Sigma Aldrich; rabbit anti-VCP (Cat# ab109240 , RRID:AB_10862588; 1:2000 for WB; 1:400 for IF), rabbit anti-LC3B (Cat# ab51520, RRID:AB_881429; 1:400 for IF), rabbit anti-GFP (Cat# ab6556, RRID:AB_305564; 1:1000), mouse anti-GFP (Cat# ab1218, RRID: AB_298911; 1:1000), rabbit anti-VPS15 (Cat# ab128903, RRID: AB_11141464; 1:1000), rabbit anti-VPS34 (Cat#

ab227861, RRID: AB_2827796; 1:1000), rabbit-anti-BiP (Cat# ab21685, RRID: AB_2119834; 1:1000), mouse-anti-GAPDH (Cat# ab8245,RRID: AB_2107448; 1:1000), rabbit anti-UFD1L (Cat# ab96648, RRID: AB_10678868; 1:1000), rabbit anti-NPL4 (Cat# ab101226, RRID:AB_10862595; 1:500), rabbit anti-CALNEXIN (Cat# ab10286, RRID:AB_2069009; 1:2000), rabbit anti-ATG7 (Cat# ab133528, RRID:AB_2532126; 1:1000), mouse anti-WIPI2 (Cat# ab105459, RRID:AB_10860881; 1:400) from Abcam; rabbit anti-LC3B (Cat# NB100-2220, RRID: AB_10003146; 1:1000), rabbit anti-TEX264 (Cat# NBP1-89866, RRID:AB_11009420; 1:1000) from Novus Biologicals; rabbit anti-BECLIN 1 (Cat# 3738, RRID: AB_490837; 1:1000), rabbit anti-K48-linkage polyubiquitin (Cat# 8081, RRID:AB_10859893, 1:1000), rabbit anti-phospho-eIF2alpha (Ser51) (Cat# 9721, RRID:AB_330951, 1:1000), rabbit anti-eIF2aplha (Cat# 9722, RRID:AB_2230924, 1:1000), rabbit anti-ATG16L1 (Cat# 8089, RRID:AB_10950320; 1:1000 for WB, 1:400 for IF) from Cell Signalling; rabbit anti-ATG14L (Cat# PD026, RRID: AB_1953054; 1:1000), mouse anti-ATG14L (Cat# M184-3, RRID: AB_10897331; 1:1000), rabbit anti-p62 (Cat# PM045, RRID:AB_1279301; 1:2000) from MBL; and mouse-anti-SCD1 from ATS bio (Cat# AB-259, RRID: AB_888013; 1:1000); mouse anti-puromycin (Cat# MABE343, RRID:AB_2566826; 1:1000), mouse anti-Huntingtin (Cat# MAB2166, RRID:AB_2123255; 1:1000), mouse anti-Polyglutamine-Expansion (Cat# MAB1574, RRID:AB_94263; 1:1000), mouse anti-Ataxin 3 (Cat# MAB5360, RRID:AB_2129339; 1:1000) from Millipore; mouse anti- Mono- and polyubiquitinylated conjugates (FK2) (Cat# BML-PW8810, RRID:AB_10541840; 1:400 for IF) from EnzoLifeSciences; mouse-anti-PI(3)P antibody (Cat# Z-P003, RRID:AB_427221) from Echelon Biosciences.
Secondary Antibodies: goat-anti-mouse Alexa Fluor 555 (Cat#A21147; 1:400), goat-anti-rabbit Alexa Fluor 488 (Cat # A32731; 1:400), streptavidin-Alexa Fluor 488 (Cat #S11223; 1:2000) from ThermoFisher.

Validation

mouse anti-Flag M2 (Cat# F1804, RRID:AB_262044), rabbit anti-Actin (Cat# A2066,RRID AB_476693), rabbit anti-GFP (Cat# ab6556, RRID:AB_305564), mouse anti-GFP (Cat# ab1218, RRID: AB_298911), mouse anti-GAPDH (Cat# ab8245, RRID: AB_2107448) were validated by the manufacturers.
rabbit anti-VCP (Cat# ab109240 , RRID:AB_10862588), rabbit anti-UFD1L (Cat# ab96648,RRID: AB_10678868), rabbit anti-NPL4 (Cat# ab101226,RRID:AB_10862595), rabbit anti-BECLIN 1 (Cat# 373,RRID: AB_490837), rabbit anti-ATG16L1 (Cat# 8089, RRID:AB_10950320), rabbit anti-ATG7 (Cat# ab133528, RRID:AB_2532126), were validated by using siRNA-mediated knockdown or CRISPR/Cas9 mediated knockout in this publication.
rabbit anti-K48-linkage polyubiquitin (Cat# 8081, RRID:AB_10859893), rabbit anti-Akt (RRID:AB_329827), rabbit anti-phospho-Akt-Ser473 (RRID:AB_2315049), rabbit anti-phospho-Akt-Thr308 (RRID:AB_329828) , mouse-anti-PI(3)P antibody (Cat# Z-P003, RRID:AB_427221), rabbit anti-LC3B (RRID: AB_10003146; 1:400 for IF), rabbit anti-LC3B (RRID: AB_10003146; 1:1000), mouse anti-WIPI2 (RRID:AB_10860881), rabbit anti-p62 (Cat# PM045, RRID:AB_1279301), rabbit anti-phospho-eIF2alpha (Ser51) (Cat# 9721, RRID:AB_330951), rabbit anti-eIF2aplha (Cat# 9722, RRID:AB_2230924) were validated by specific treatment in this publication (for example MG132 treatment or EBSS starvation treatment).
mouse anti-Huntingtin (Cat# MAB2166, RRID:AB_2123255), mouse anti-Polyglutamine-Expansion Milipore (Cat# MAB1574, RRID:AB_94263) and mouse anti-Ataxin 3 Milipore (Cat# MAB5360, RRID:AB_2129339) were validated using control patient fibroblasts in this publication.
rabbit anti-VPS15 (Cat# ab128903, RRID: AB_11141464; 1:1000), rabbit anti-VPS34 (Cat# ab227861,RRID: AB_2827796; 1:1000), rabbit anti-ATG14L (Cat# PD026, RRID: AB_1953054; 1:1000), mouse anti-ATG14L (Cat# M184-,RRID: AB_10897331; 1:1000) were validated in Immunoprecipitation experiments in this publication.
mouse anti-puromycin (Cat# MABE343, RRID:AB_2566826; 1:1000) was validated using puromycin non-treated samples (data not shown).

Eukaryotic cell lines

Policy information about cell lines

Cell line source(s)

human cervical epithelium HeLa cell line (ATCC #CCL-2; CVCL_0030)
human embryonic kidney HEK293 cell line (ECACC #85120602)
wild-type HTT Q7/Q7 striatal cell line (Coriell Institute #CH00097)
homozygous HTT Q111/Q111 striatal cell line (Coriell Institute #CH00095)
human embryonic suspension cells (Expi293F Gibco #A14527)
human neuroblastoma SH-SY5Y cell line (ECACC #94030304)
unaffected fibroblast control (1) (Coriell Institute #GM04711)
unaffected fibroblast control (2) (Coriell Institute #GM04729)
Huntington's disease patient fibroblasts (HTTpolyQ17/45) (Coriell Institute #GM04476)
Huntington's disease patient fibroblasts (HTTpolyQ15/70) (Coriell Institute #GM21756)
Huntington's disease patient fibroblasts (HTTpolyQ15/66) (Coriell Institute #GM21757)
Huntington's disease patient fibroblasts (HTTpolyQ17/80) (gift from Ferdinando Squitieri, Huntington and Rare Diseases Unit, Fondazione IRCCS Casa Sollievo della Sofferenza Research Hospital, Italy #30501)
spinocerebellar ataxia type 3 patient fibroblasts (Coriell Institute #GM06153)
HeLa TALEN BECLIN1 knockout control cell line (gift from Wensheng Wei, Peking University, Beijing He et al., 2015)
HeLa TALEN BECLIN1 knockout cell line (gift from Wensheng Wei, Peking University, Beijing He et al., 2015)
HeLa CRISPR/Cas9 ATG16L1 knockout control cell line (Bento et al., 2016a)
HeLa CRISPR/Cas9 ATG16L1 knockout cell line (Bento et al., 2016a)
Ub-G76V-GFP HeLa cell line (Korolchuk et al., 2009)
SRAI-LC3B reporter cell line (generated in this study)
EGFP-α-synuclein-A53T cell line (generated in this study)
EGFP-α-synuclein-A53T ATG7 knockout cell line (generated in this study)

Authentication

HeLa authenticated by ATCC (DNA barcoding method; FTA Barcode: STRA1466)
HEK293 purchased from ECACC and authenticated by ECACC (DNA profile, Karyotype)
SH-SY5Y purchased from ECACC and authenticated by ECACC (DNA profile, Karyotype)
Expi293F purchased from Gibco and authenticated by Gibco(DNA profile, Karyotype)
BECLIN1 KO cells were authenticated by Western blot analysis with Beclin1 antibody

ATG16 KO cells were authenticated by Western blot analysis with Atg16 antibody
ATG7 KO cells were authenticated by Western blot analysis with Atg7 antibody
HTT Q7/Q7 and Q111/Q111 striatal cell lines were authenticated by Western blot analysis with Huntingtin antibody (different migration of Huntingtin protein caused by the presence of polyQ expansion) and polyQ antibody
Huntington's disease patient fibroblasts were authenticated by Western blot analysis with Huntingtin antibody (different migration of Huntingtin protein caused by the presence of polyQ expansion) and polyQ antibody
spinocerebellar ataxia type 3 patient fibroblasts were authenticated by Western blot analysis with Ataxin3 antibody (different migration of Ataxin3 protein caused by the presence of polyQ expansion)
Ub-G76V-GFP HeLa cells were authenticated by assessing their FACS profile and by Western blot detection of Ub-G76V-GFP
SRAI-LC3B HeLa reporter cells were authenticated by assessing their FACS profile
EGFP-α-synuclein-A53T HeLa cells were authenticated by Western blot analysis with GFP and synuclein specific antibody

Mycoplasma contamination	The cells were regularly tested using EZ-PCR Mycoplasma Test Kit (Biological Industries; cat#20-700-20). Cells used in this study were mycoplasma negative.
Commonly misidentified lines (See ICLAC register)	no commonly misidentified cell lines were used in the study

Flow Cytometry

Plots

Confirm that:

☒ The axis labels state the marker and fluorochrome used (e.g. CD4-FITC).

☒ The axis scales are clearly visible. Include numbers along axes only for bottom left plot of group (a 'group' is an analysis of identical markers).

☒ All plots are contour plots with outliers or pseudocolor plots.

☒ A numerical value for number of cells or percentage (with statistics) is provided.

Methodology

Sample preparation	HeLa cells stably expressing GFP-tagged mutant αsynuclein (EGFP-A53T), Ub-G76V-GFP or SRAI-LC3B cells were treated with various compounds for 24 h. Cells were then trypsinized and fluorescence was analysed using an Attune NxT Flow Cytometer (ThermoFisher Scientific) using the BL1 (488 530/30) detector (for GFP-tagged mutant αsynuclein) or using the VL2 (405 512/25) and BL1 (488 530/30) detectors (for SRAI-LC3B).
Instrument	Attune NxT Flow Cytometer (ThermoFisher Scientific)
Software	FlowJo software (Three Star)
Cell population abundance	All cells are stably expressing indicated protein with EGFP or TOLLES-YPet tag.
Gating strategy	Cells were first gated on forward (FSC-A) and side scatter (SSC-A) for P1 and then for singlets (FSC-A/FSC-H) for P2. 20,000 single cells were recorded for each replicate. GFP+ gates were set using normal HeLa cells. For SRAI-LC3B cells the ratio of VL2 to BL1 signals was derived for each cell and the median ratio per condition was used for analysis.

☒ Tick this box to confirm that a figure exemplifying the gating strategy is provided in the Supplementary Information.
